# PNO1, which is negatively regulated by miR-340-5p, promotes lung adenocarcinoma progression through Notch signaling pathway

**DOI:** 10.1038/s41389-020-0241-0

**Published:** 2020-06-01

**Authors:** Dongming Liu, Li Lin, Yajie Wang, Lu Chen, Yuchao He, Yi Luo, Lisha Qi, Yan Guo, Liwei Chen, Zhiqiang Han, Guangtao Li, Qiang Li, Zhiyong Liu, Peng Chen, Hua Guo

**Affiliations:** 1 Department of Tumor Cell Biology, Tianjin Medical University Cancer Institute and Hospital, Tianjin, 300060 China; 2 Department of Hepatobiliary Cancer, Liver Cancer Research Center, Tianjin Medical University Cancer Institute and Hospital, Tianjin, 300060 China; 3 National Clinical Research Center for Cancer, Key Laboratory of Cancer Prevention and Therapy, Tianjin’s Clinical Research Center for Cancer, Tianjin, 300060 China; 4 Department of Thoracic Oncology, Lung Cancer Diagnosis and Treatment Center, Tianjin Medical University Cancer Institute and Hospital, Tianjin, 300060 China; 5 Department of Pathology, Tianjin Medical University Cancer Institute and Hospital, Tianjin, 300060 China; 6 Cancer Biobank of Tianjin Medical University Cancer Institute and Hospital, Tianjin, 300060 China

**Keywords:** Non-small-cell lung cancer, Tumour biomarkers

## Abstract

Many studies have shown that the hyperactivation of ribosome biogenesis plays essential roles in the initiation and progression of cancers. As a ribosome assembly factor, PNO1 plays an important role in ribosome biogenesis. However, little is known about the expression and function of PNO1 in human tumors. In our present study, we aimed to explore the functional roles and the underlying molecular mechanisms of PNO1 in human lung adenocarcinoma (LUAD). Both bioinformatics databases and tumor tissues demonstrated that the expression of PNO1 in LUAD tissues was higher than that in adjacent tissues and predicted poor survival in LUAD patients. In vitro and in vivo assays suggested that downregulation of PNO1 expression suppressed LUAD cell proliferation and invasion. Further studies found that miR-340-5p depressed PNO1 expression via direct binding to the 3′ untranslated region (UTR) of PNO1. PNO1 expression was negatively correlated with miR-340-5p expression in LUAD cells and tissue samples. Moreover, upregulation or downregulation of miR-340-5p expression reversed the effects of PNO1 inhibition and overexpression, respectively. Meanwhile, downregulation of PNO1 inhibited Notch signaling pathway which modulated epithelial mesenchymal transition (EMT). These results indicate that PNO1, negatively regulated by miR-340-5p, played an important role in LUAD progression via Notch signaling pathway. The miR-340-5p/PNO1/Notch axis might be a potential target for individualized and precise treatment of LUAD patients in the future.

## Introduction

Lung cancer is the leading cause of cancer-related death worldwide^1,2^. Approximately 85% of lung cancer cases are histologically classified as non-small cell lung cancer (NSCLC); the rest are classified as small cell lung cancer^3^. Lung adenocarcinoma (LUAD) represents the predominant histological phenotype of NSCLC^4^. The predicted 5-year survival rate of LUAD patients is nearly 20%^5,6^. What causes the poor prognosis of LUAD patients?

On the one hand, for most LUAD patients with early stage disease who undergo surgery, a lack of specific prognostic biomarkers is the main factor that restricts postoperative disease monitoring^7^. On the other hand, the main oncogenic drivers in NSCLC patients (especially individuals with LUAD) such as mutations in EGFR, KRAS and BRAF, translocations of ROS1 and RET and ALK rearrangements are well known^8^. There is still a large proportion of LUAD patients who cannot benefit from current targeted therapies^9^. The main reason for this phenomenon is the lack of novel effective targets^10^. Thus, when researchers fully understand the high degree of molecular heterogeneity inherent in LUAD, especially the diversity of carcinogenic driver genes, LUAD patients will benefit from precise individualized treatment^11,12^. Overall, the exploration of novel prognostic biomarkers and effective therapeutic targets is important for the control of LUAD.

Ribosomes are produced in the nucleolus (the most important nuclear substructure)^13^. In eukaryotes, ribosomes act as molecular machines in cells and are responsible for translating mRNA into protein^14^. In many malignant tumors, such as lung cancer, liver cancer and breast cancer tumors, increased numbers of nucleoli and larger nucleoli are frequently observed, which leads to an increased rate of ribosomal biogenesis^15^. These important observations demonstrate that the hyperactivation of ribosome biogenesis plays essential roles in the initiation and progression of cancers, including lung cancer^16,17^. Obviously, inhibiting ribosome biogenesis might provide a new therapeutic strategy for cancer treatment^14^.

The RNA-binding protein “partner of NOB1” (PNO1) is known to be a ribosome assembly factor and is essential in ribosome biogenesis^18–20^. PNO1, which is also called Dim2 or Rrp20, is highly conserved from yeast to mammals^21^. PNO1 is responsible for the cleavage of 18S mediated by binding to NOB1^22^. Deletion of PNO1 leads to the inhibition of 18S synthesis and a decrease in 40S subunit synthesis^19^. In contrast to the extensive research on the ribosome-related function of PNO1, little is known about the role of PNO1 in cancer cells. Only one study demonstrated the critical function of PNO1 in ribosome biogenesis in human colorectal cancer (CRC) cells. In addition, PNO1 can be used as a potential biomarker in CRC^20^. Therefore, exploring the possibility of PNO1 as a prognostic biomarker and fully understanding the function of PNO1 in cancer progression are crucial for LUAD patients.

In this study, by using database exploration of The Cancer Genome Atlas (TCGA) and Gene Expression Omnibus (GEO) followed by immunohistochemical (IHC) staining validation of LUAD and lung squamous cell carcinoma (SCLC) patient samples, we confirmed that high expression of PNO1 was intently related to the poor prognosis of LUAD. More importantly, PNO1 might serve as a specific prognostic biomarker in LUAD patients. Downregulation of PNO1 expression suppressed the proliferation and metastasis of LUAD cells in in vitro functional experiments. Furthermore, we demonstrated that miR-340-5p directly targeted PNO1 by using a luciferase reporter assay. In a series of rescue experiments, the results further indicated that the functional roles of PNO1 might be regulated by miR-340-5p expression levels in LUAD. In addition, PNO1 expression was correlated with Notch pathway and epithelial mesenchymal transition (EMT) through GSEA datasets, Western Blotting, RT-PCR and some functional experiments. We assumed that PNO1 promoted LUAD progression via Notch signaling. Finally, a xenograft model and lung metastasis assay showed that high expression of PNO1 might promote the proliferation and metastasis of LUAD in vivo. In summary, our research might provide an attractive therapeutic strategy (such as exploitation of miR-340-5p or Notch inhibitor) for targeting PNO1 in LUAD.

## Results

### PNO1 expression is upregulated in LUAD tissue samples and predicts poor survival in LUAD patients

First, we used bioinformatic methods to explore the relationship between PNO1 expression and LUAD patient prognosis. Microarray analyses (TCGA, GSE40791, GSE7670, GSE33532, GSE101929, GSE32863, GSE10072, GSE21933, and GSE68571 datasets) indicated that PNO1 expression was upregulated in LUAD samples and was associated with advanced TNM stages and lymph node metastasis (Supplementary Fig. 1A–D). In addition, based on detailed survival information from the TCGA and GEO database, the cutoff value for PNO1 was set by the quartile or median method for further survival analysis. The results revealed that the high expression of PNO1 was positively correlated with the poor prognosis of LUAD patients, such as overall survival (OS) and disease-free survival (DFS) (*P* = 0.082 and *P* = 0.036 on TCGA datasets, *P* = 0.046 on GSE68571 respectively, Supplementary Fig. 1E–G). Notably, PNO1 expression was also upregulated in SCLC and NSCLC samples (Supplementary Fig. 2A, D). However, there were no significant associations between the expression level of PNO1 and the prognosis (OS and DFS) of SCLC and NSCLC patients (SCLC: *P* = 0.050 and *P* = 0.055, respectively, Supplementary Fig. 2B, C; NSCLC: *P* = 0.546 and *P* = 0.255, respectively, Supplementary Fig. 2E, F). The results suggested that there was a unique relationship between the expression of PNO1 and the prognosis of LUAD patients, which was further validated by IHC staining of clinical samples. Based on mRNA expression data from the GSE7670 and GSE40791 samples evaluated by GSEA, we confirmed that low PNO1 expression was correlated with good survival in LUAD patients (*P* < 0.0001 and *P* = 0.028, Supplementary Fig. 1H, I). Furthermore, we found that PNO1 may be related to gefitinib chemotherapy resistance through GSE40791, which also showed that it could trigger the poor prognosis (*P* = 0.039, Supplementary Fig. 1J). In addition, the GSEA datasets demonstrated that high expression of PNO1 was correlated with LUAD cell proliferation and metastasis (Supplementary Fig. 1K–M). Finally, through the pancancer view of UALCAN (http://ualcan.path.uab.edu/index.html), PNO1 expression was found to be upregulated in most types of cancers (Supplementary Fig. 2G).

### PNO1 is a specific prognostic biomarker in LUAD

**Fig. 1:**
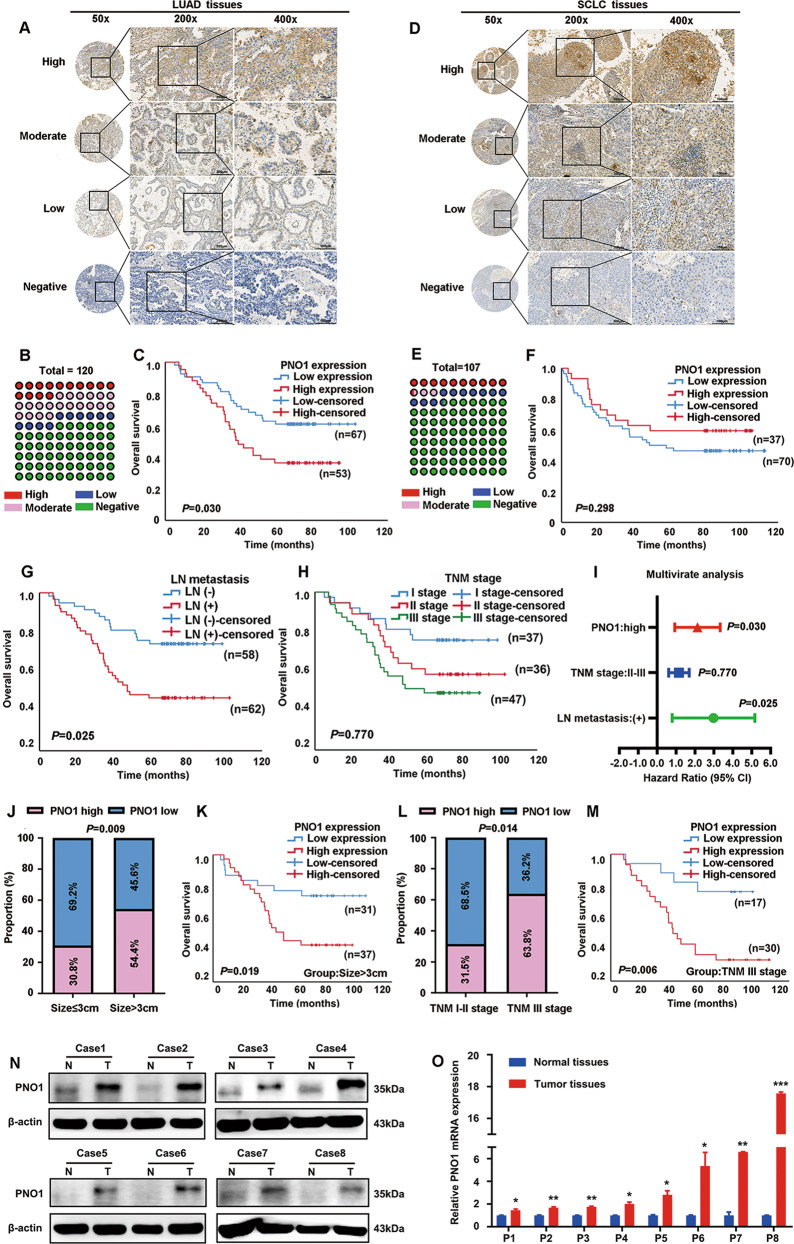
PNO1 is a specific prognostic biomarker in LUAD. **a** IHC analysis of PNO1 expression in tissue microarrays of LUAD specimens (scale bar, 200 μm or 100 μm). **b** The proportions of LUAD patients with different PNO1 expression levels. **c** Kaplan–Meier survival curve showing the correlation between the PNO1 IHC score and OS of LUAD patients. **d** IHC analysis of PNO1 expression in tissue microarrays of SCLC specimens (scale bar, 200 μm or 100 μm). **e** The proportions of SCLC patients with different PNO1 expression levels. **f** Kaplan–Meier survival curve analysis showing the correlation between the PNO1 IHC score and OS in SCLC patients. **g**, **h** Kaplan–Meier survival curve analysis showing the correlations between LN metastasis or TNM stage and OS in LUAD patients. **i** Forest plots showing the multivariate risk factors of LUAD patients. **j** Correlation analysis between the PNO1 IHC score and tumor size. **k** Kaplan-Meier survival curve showing the correlation between the PNO1 IHC score and OS in a subgroup of LUAD patients (tumor size >3 cm). **l** Correlation analysis between the PNO1 IHC score and TNM stage. **m** Kaplan–Meier survival curve showing the correlation between the PNO1 IHC score and OS in a subgroup of LUAD patients (TNM stage III). **n**, **o** Western Blotting and RT-PCR showing the differences in the PNO1 protein and mRNA levels between eight pairs of LUAD tissue and adjacent nonmalignant tissue. nsP > 0.05, *P < 0.05, **P < 0.01, ***P < 0.001.

To further probe the expression pattern of PNO1 in LUAD, a large cohort of 120 LUAD samples was evaluated by IHC staining. Representative photomicrographs of different PNO1 expression levels are shown in Fig. 1a. More than half of the LUAD patient samples had negative PNO1 staining (Fig. 1b). The Cox regression model showed that increased expression of PNO1 (Fig. 1c) was an independent risk factor for OS in LUAD (Supplementary Table 1). Patients with low PNO1 expression had a 5-year OS rate of 68.7%, which was much better than that of 49.1% for patients with high PNO1 expression (Supplementary Table 1). To verify the results (Supplementary Fig. 2B, C) for the TCGA database (SCLC), we also examined PNO1 expression in 107 SCLC patients using immunohistochemistry. Representative photomicrographs were shown in Fig. 1d. Surprisingly, nearly 75% of the SCLC patients had negative PNO1 staining (Fig. 1e). Similarly, there was no significant association between PNO1 expression and OS in SCLC (*P* = 0.298, Fig. 1f). In addition, LN metastasis was another independent risk factor for LUAD patients (Fig. 1g). While, TNM stage (*P* = 0.770) was not the independent risk factor (Fig. 1h). In summary, multivariate analysis results for LUAD patients were presented in the forest plots (Fig. 1i). This series of results indicated that PNO1 could be used as a specific biomarker in LUAD patients.

Next, associations of PNO1 expression with various clinicopathological factors were assessed. We found that the expression level of PNO1 was closely correlated with tumor size and TNM stage (Supplementary Table 2). In total, 37 out of 68 (54.4%) cases with a tumor size >3 cm showed high PNO1 expression. In contrast, only 16 out of 52 (30.8%) cases with a tumor size ≤ 3 cm exhibited high PNO1 expression (*P* = 0.009, Fig. 1j, Supplementary Table 2). Furthermore, among 73 LUAD patients with TNM stage I or II disease, 50 (68.5%) had low PNO1 expression; however, among LUAD patients with advanced stage III disease, the percentage of patients with low PNO1 expression was only 36.2% (17/47) (*P* = 0.014, Fig. 1l, Supplementary Table 2). This finding also coincided with the results for the TCGA and GSE40791 datasets (Supplementary Fig. 1B, C). Remarkably, tumor size and TNM stage were the most critical independent risk factors for a poor prognosis in LUAD^23^. Then, we further probed the differential prognosis between LUAD patients with high or low PNO1 expression in two poor prognosis subgroups: tumor size > 3 cm and TNM stage III disease. Interestingly, in these two subgroups, the patients with low expression levels of PNO1 could still achieve longer OS than those in the high expression cohort (*P* = 0.019, *P* = 0.006, Fig. 1k, m). In addition, we also examined the PNO1 expression and survival rate of LN (+) patients. The survival cures showed that high PNO1 expression was related bad prognosis of LN (+) patients (Supplementary Fig. 2H). Then, we examined the protein expression level of PNO1 in 8 pairs of fresh LUAD tumor tissue samples and the corresponding nonmalignant tissue samples by Western Blotting. The protein level of PNO1 in the LUAD tissue samples was considerably higher than that in the adjacent normal tissue samples (Fig. 1n). Furthermore, similar results were identified by RT-PCR (Fig. 1o).

### Knocking down PNO1 expression suppresses LUAD cell migration and invasion in vitro

**Fig. 2:**
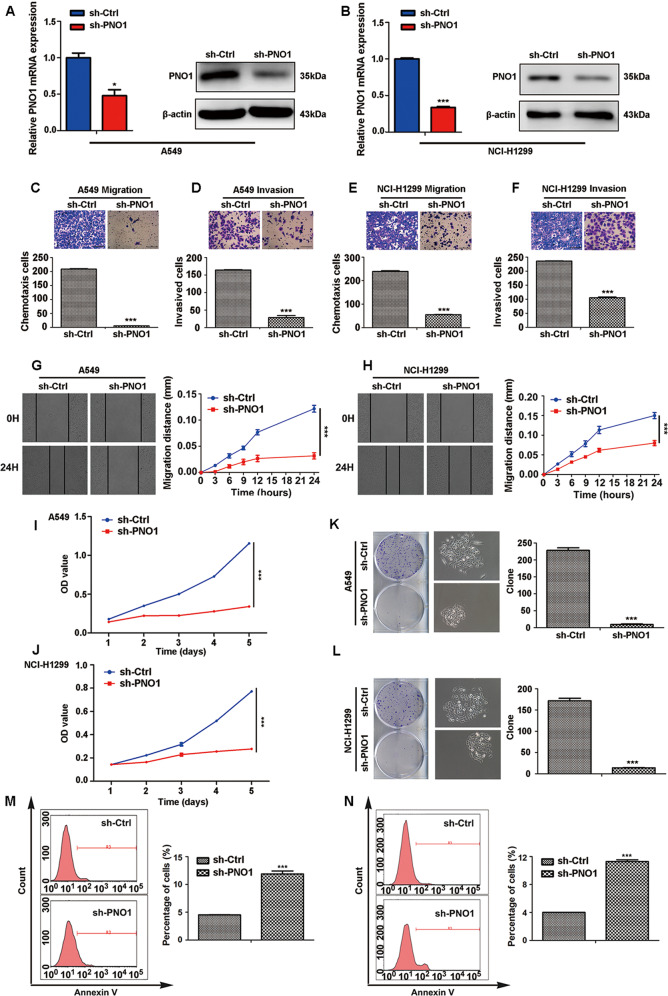
Knocking down PNO1 expression suppresses LUAD cell metastasis and proliferation in vitro. **a**, **b** The construction of PNO1-control (sh-Ctrl) and PNO1-knockdown (sh-PNO1) A549 and NCI-H1299 LUAD cell lines. **c**, **d** The chemotaxis potential and invasive ability of the A549 sh-Ctrl and sh-PNO1 groups (scale bar, 1.0 mm). **e**, **f** The chemotaxis potential and invasive ability of the NCI-H1299 sh-Ctrl and sh-PNO1 groups (scale bar, 1.0 mm). **g**, **h** Wound-healing assay comparing the migration distance between the sh-Ctrl and sh-PNO1 groups of A549 or NCI-H1299 cells. **i**, **j** MTT assay showing the proliferative abilities in the sh-Ctrl and sh-PNO1 groups of A549 or NCI-H299 cells. **k**, **l** Comparison of the proliferative potential between the sh-Ctrl and sh-PNO1 groups of A549 or NCI-H299 cells by a colony formation assay. **m**, **n** Cell apoptosis assay revealing the proportions of apoptotic A549 and NCI-H1299 cells in the sh-PNO1 and sh-Ctrl groups. *P < 0.05, **P < 0.01, ***P < 0.001.

To further investigate the role of PNO1 in the tumor progression of LUAD, we selected two wild-type LUAD cell lines (A549 and NCI-H1299) to construct cell lines with stable PNO1 downregulation (sh-PNO1) and PNO1 upregulation (PNO1) via transfection with lentiviral plasmids. These two wild-type cell lines were also transduced with an empty vector as a control (sh-Ctrl or Vector). The efficiencies of PNO1 deletion and overexpression were confirmed by Western Blotting and RT-PCR (Fig. 2a, b, Supplementary Fig. 3A, B).

To probe the biological role of PNO1 in regulating LUAD progression, GSEA was performed, and the results indicated that PNO1 was positively correlated with tumor metastasis based on PNO1 mRNA levels in the GSE40791 datasets (Supplementary Fig. 1L, M). Next, we further investigated the biological functions of PNO1 in A549 and NCI-H1299 cell metastasis. Migration and invasion assays verified that the downregulation of PNO1 expression suppressed the migration and invasion abilities of A549 and NCI-H1299 cells, respectively (Fig. 2c–f). The wound-healing assay (Fig. 2g, h) also confirmed that PNO1 promoted the ability of nondirectional migration in both A549 and NCI-H1299 cells.

In addition, we found that the overexpression of PNO1 promoted the metastasis abilities of A549 and NCI-H1299 cells by invasion assay (Supplementary Fig. 3C, D). Similarly, the upregulation of PNO1 expression accelerated the nondirectional migration ability of A549 and NCI-H1299 cell lines through the wound-healing assay (Supplementary Fig. 3E, F).

### PNO1 promotes LUAD cell proliferation and inhibits cell apoptosis

First, GSEA indicated that PNO1 was positively associated with tumor cell proliferation (Supplementary Fig. 1K). The proliferation-related genes in stem cell-like phenotypes was positively correlated with ‘PNO1_positive’. Compared with sh-Ctrl treatment, knocking down PNO1 expression significantly suppressed LUAD cell proliferation based on cell proliferation (Fig. 2i, j) and colony formation assays (Fig. 2k, l) with A549 and NCI-H1299 cells. On the other hand, compared with Vector treatment, PNO1 overexpression significantly promoted LUAD cell proliferation based on CCK-8 assay (Supplementary Fig. 3G, H). A cell apoptosis assay revealed that the proportions of apoptotic A549 and NCI-H1299 cells in the sh-PNO1 groups were higher than those in the sh-Ctrl groups (Fig. 2m, n). We also evaluated the apoptosis by Annexin V/PI assay. The result showed that the proportions of early and apoptotic A549 cell in the sh-PNO1 groups were higher than those in the sh-Ctrl groups (Supplementary Fig. 3I, J).

### miR-340-5p directly targets PNO1, whose expression is specifically downregulated in LUAD patients

**Fig. 3:**
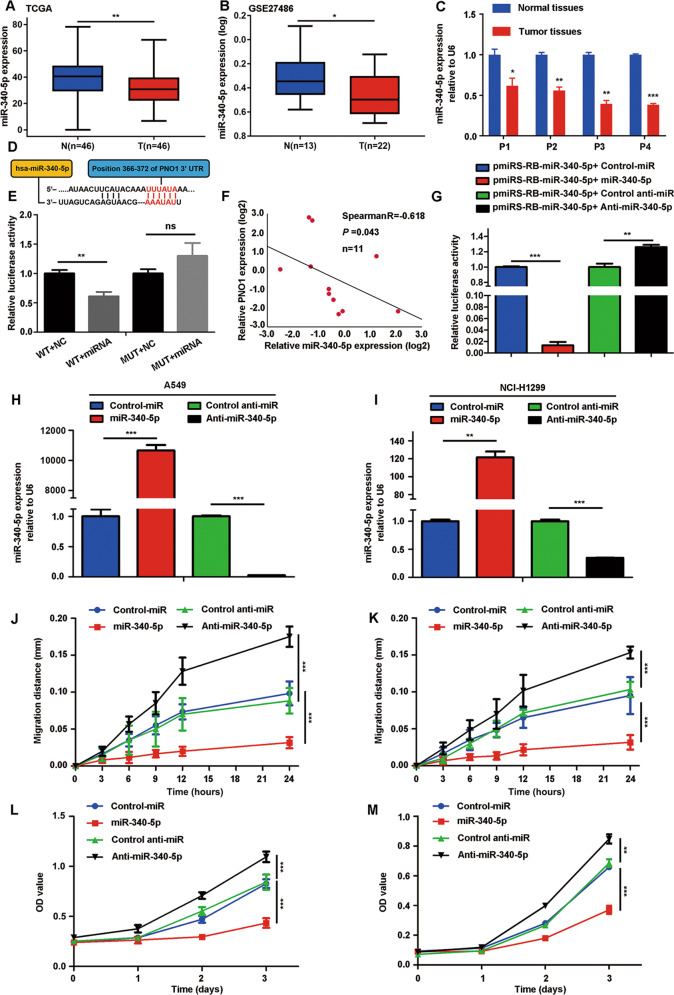
miR-340-5p directly targets PNO1, whose expression is specifically downregulated in LUAD patients. **a**, **b** The expression of miR-340-5p in LUAD tissue samples and adjacent nonmalignant tissue samples from the TCGA and GSE27486 databases. **c** RT-PCR showing the differences in miR-340-5p mRNA levels between four pairs of LUAD tissue and adjacent nonmalignant tissue. **d** The sequence of the 3′ UTR of PNO1. **e** Luciferase reporter assays for PNO1 and miR-340-5p (WT, h-PNO1-WT; MUT, h-PNO1-MUT; miRNA, miR-340-5p). **f** The correlation between the expression levels of miR-340-5p and PNO1 in LUAD patients determined by RT-PCR. **g** miR-340-5p sensor luciferase reporter for measurement the functional activity of miR-340-5p. **h**, **i** Establishment of different miR-340-5p expression levels in A549 and NCI-H1299 cells. **j**, **k** Different migratory abilities associated with different expression levels of miR-340-5p in A549 and NCI-H1299 cells. **l**, **m** Different proliferative potentials associated with different expression levels of miR-340-5p in A549 and NCI-H1299 cells. *P < 0.05, **P < 0.01, ***P < 0.001. (miR-340-5p: miR-340-5p overexpression; anti-miR-340-5p: miR-340-5p downregulation; control-miR and control-anti-miR: respective control groups).

We further probed the molecular mechanism underlying PNO1-mediated LUAD proliferation and metastasis. We examined whether miRNAs could regulate PNO1 levels in LUAD patients. The method for predicting miRNAs is presented in Supplementary Fig. 4A. We then selected the intersections of different miRNAs in TargetScan (http://www.targetscan.org/vert_72/) and miRDB (http://www.mirdb.org/) that were predicted to bind to the 3′ UTR of PNO1 (Supplementary Fig. 4B, C). Since we had confirmed that PNO1 expression is upregulated in LUAD tissue, the potential upstream miRNAs should exhibit downregulated expression in LUAD samples based on the negative miRNA-mRNA regulation mechanism. We measured the expression levels of identified miRNAs (miR-340-5p, miR-6504-5p, miR-3064-3p, and miR-377-3p) by using TCGA miRNA datasets. Surprisingly, among these miRNAs, only miR-340-5p exhibited downregulated expression in LUAD tissue samples (Fig. 3a, Supplementary Fig. 4D-F). We also verified this result with GSE27486 datasets and LUAD tumor tissue samples (Fig. 3b, c). It has been reported that miR-340-5p has an antitumor effect on many cancers, including NSCLC^24,25^.

Furthermore, to confirm the relationship between miR-340-5p and the 3′ UTR of PNO1, we performed a luciferase reporter assay. The predicted binding site for miR-340-5p in PNO1 is shown in Fig. 3d. Then, we identified that a miR-340-5p mimic efficiently reduced the luciferase activity of a PNO1-3′ UTR-WT reporter in HEK293T cells but not that of a 3′ UTR-mutated PNO1 reporter, in which the predicted binding site was mutated previously (Fig. 3e). In addition, the relative expression levels of miR-340-5p and PNO1 in LUAD samples and their relationship were further verified in LUAD tissue samples by RT-PCR (Spearman *R* = −0.618, *P* = 0.043, *n* = 11) (Fig. 3f). We concluded that PNO1 was regulated by miR-340-5p in LUAD.

To further probe whether the PNO1-mediated promotion of LUAD cell proliferation and metastasis is actually regulated by miR-340-5p, we first detected the function of miR-340-5p in LUAD cells. Thus, miR-340-5p/control-miR and anti-miR-340-5p/control anti-miR lentiviral plasmids were transfected into A549 and NCI-H1299 cells, respectively. Then we used miR-340-5p sensor dual luciferase reporter to evaluate the functional activity of miR-340-5p after transient transfection in A549 cell line. We found that the relative luciferase activity in ‘pmiRS-miR-340-5p+miR-340-5p’ group was downregulation compared with ‘pmiRS-miR-340-5p+Control-miR’ group. It revealed that the functional activity of miR-340-5p in ‘pmiRS-miR-340-5p+miR-340-5p’ group was increased. On the other hand, the change of luciferase activity in ‘pmiRS-miR-340-5p+Anti-miR-340-5p’ group demonstrated the decrease in miR-340-5p functional activity compared with ‘pmiRS-miR-340-5p+Control anti-miR’ group. (Fig. 3g). The relative miR-340-5p expression in different groups of these two cells was detected by RT-PCR (Fig. 3h, i). Furthermore, we found that miR-340-5p overexpression inhibited LUAD cell migration. On the other hand, downregulation of miR-340-5p expression promoted the migration of A549 and NCI-H1299 cells (black lines in Fig. 3j, k, Supplementary Fig. 4G, H). Similarly, knocking down miR-340-5p expression promoted cell growth based on cell proliferation (black lines in Fig. 3l, m) and colony formation assays (Supplementary Fig. 4I, J). In contrast, we also found that overexpressing miR-340-5p suppressed cell proliferation in A549 and NCI-H1299 cells (red lines in Fig. 3l, m, Supplementary Fig. 4I, J).

**Fig. 4:**
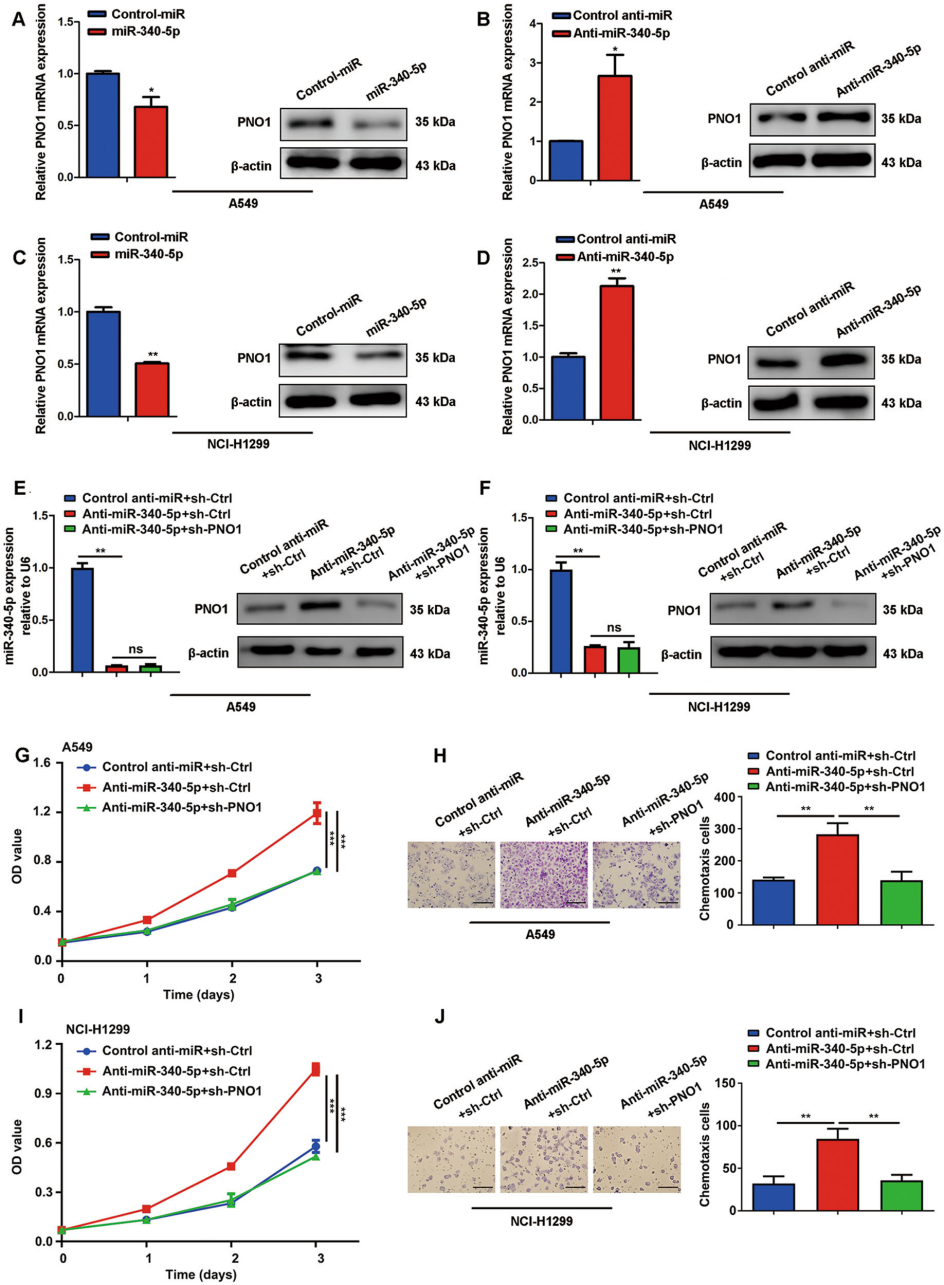
The functional roles of PNO1 are regulated by miR-340-5p expression levels in LUAD. **a**–**d** The verification of the expression level of a target gene (PNO1) by Western Blotting and RT-PCR in A549 and NCI-H1299 cell lines. **e**, **f** The expression of miR-340-5p and PNO1 in control anti-miR+sh-Ctrl, anti-miR-340-5p+sh-Ctrl and anti-miR-340-5p+sh-PNO1 groups of A549 and NCI-H1299 cells. **g**–**j** Different proliferative potentials and migratory abilities associated with different expression levels of miR-340-5p and PNO1 in A549 and NCI-H1299 cells. *P < 0.05, **P < 0.01, ***P < 0.001.

To determine the regulation of PNO1 by miR-340-5p, we detected PNO1 mRNA and protein expression in stable miR-340-5p/control-miR-transfected cells and anti-miR-340-5p/control anti-miR-transfected cells. Obviously, PNO1 expression was downregulated in the miR-340-5p-overexpressing cell lines, but PNO1 was highly expressed in the anti-miR-340-5p-expressing cells compared with the control cells (Fig. 4a–d). Moreover, we transfected the anti-miR-340-5p lentiviral plasmid followed by a construct for PNO1 downregulation (sh-PNO1) into A549 and NCI-H1299 cells. We then examined the expression levels of miR-340-5p and PNO1 (Fig. 4e, f). Interestingly, downregulation of PNO1 expression (sh-PNO1 transfection) attenuated the promotion of growth and migration induced by anti-miR-340-5p, as detected by cell proliferation and chemotaxis assays with A549 and NCI-H1299 cells (Fig. 4g–j). We also transfected PNO1 plasmid and control vector into miR-340-5p overexpressed A549 cells to get ectopic PNO1 expression cells (miR-340-5p + PNO1) and control cells (miR340-5p+Vector). The RT-PCR results indicated that miR-340-5p level was increased in miR-340-5p + PNO1 and miR340-5p+Vector groups compared to Control miR+Vector group. (Supplementary Fig. 4K). On the other hand, the protein expression of PNO1 was increased in miR-340-5p + PNO1 group compared with miR-340-5p+Vector group (Supplementary Fig. 4L). In addition, upregulation of PNO1 expression partially attenuated the inhibition of growth and migration induced by miR-340-5p, as detected by colony formation and wound-healing assays with A549 cell (Supplementary Fig. 4M, N). In summary, these results indicated that the functional roles of PNO1 might be regulated by miR-340-5p expression levels in LUAD.

### Downregulation of PNO1 inhibits LUAD progression through Notch pathway and EMT

**Fig. 5:**
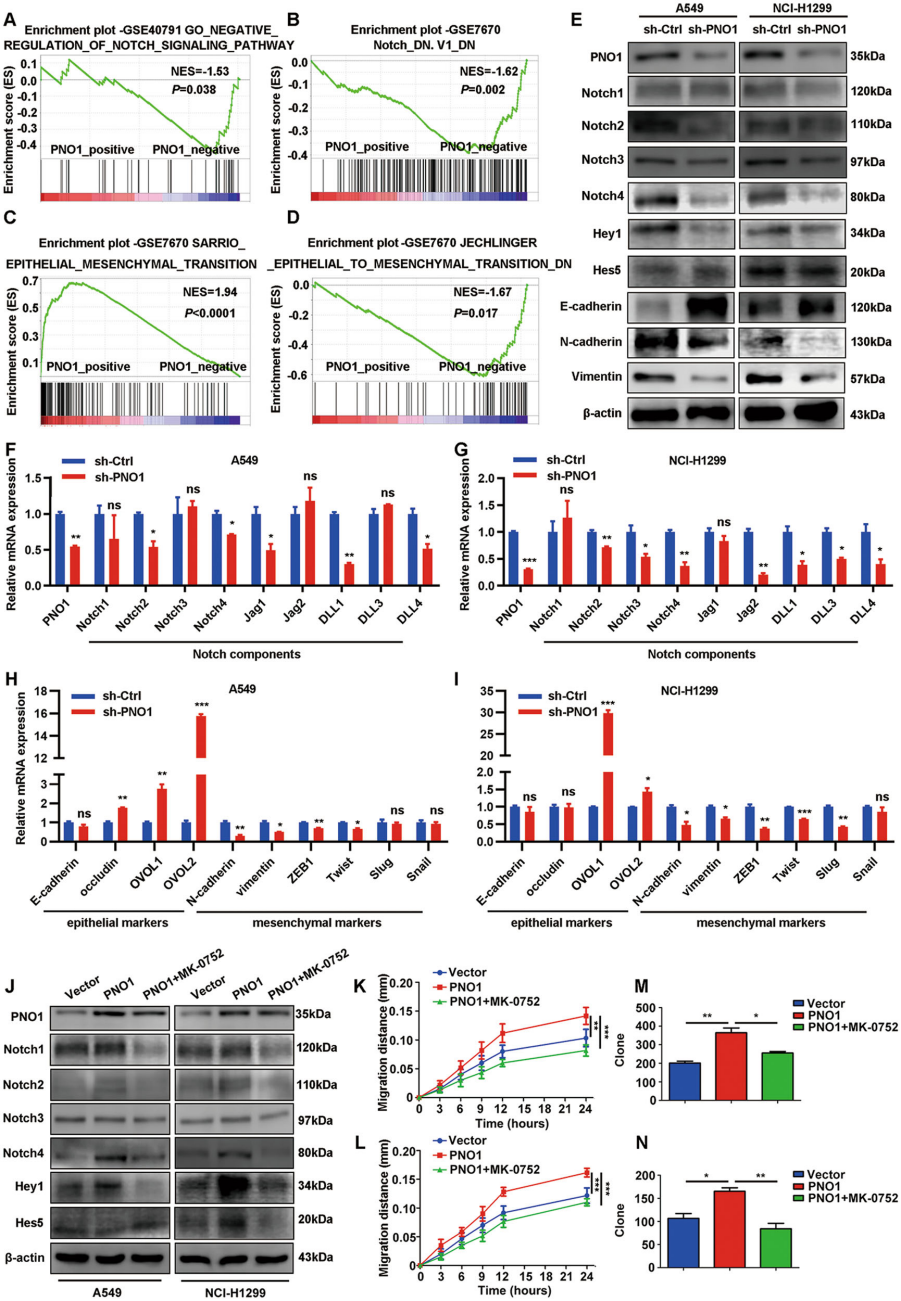
Downregulation of PNO1 inhibits LUAD progression through Notch pathway and EMT. **a**, **b** GSEA showed the relationship between the expression of PNO1 and Notch signaling pathway. **c**, **d** The correlation of PNO1 with EMT was investigated by GSEA analysis on the basis of GSE7670 datasets. **e** The protein level of each Notch intracellular domain, Notch target genes or EMT markers (such as Notch1-4, Hey1, Hes5, E-cadherin, N-cadherin, and Vimentin) was assessed in sh-Ctrl and sh-PNO1 groups by Western Blotting. **f**, **g** The expression of Notch components (such as Notch2, Notch4, DLL1, and DLL4) was downregulated in sh-PNO1 groups of A549 or NCI-H299 cells by RT-PCR assay. **h**, **i** Epithelia-associated transcription factors (such as OVOL1 and OVOL2) expression was upregulated and mesenchymal-associated transcription factors (such as N-cadherin, vimentin, ZEB1, and Twist) expression was downregulated in the sh-PNO1 groups of A549 and NCI-H1299 by RT-PCR assay. **j** The protein level of each Notch intracellular domain and Notch target genes (such as Notch1-4, Hey1, and Hes5) was assessed in Vector, PNO1 or PNO1 + MK-0752 groups by Western Blotting. **k**, **l** The migration potential detected by wound-healing assay in Vector, PNO1 or PNO1 + MK-0752 groups. **m**, **n** The proliferation potential detected by colony formation assay in Vector, PNO1 or PNO1 + MK-0752 groups. nsP > 0.05, *P < 0.05, **P < 0.01, ***P < 0.001.

To further demonstrate the downstream molecular mechanism of PNO1, we used GSEA software to explore some related signaling pathway. The results about GSEA datasets indicated that PNO1 might be as an oncogene promoted LUAD progression through Notch signaling pathway (Fig. 5a, b). Previous studies report that Notch signaling pathway participates the establishment of invasive mesenchymal phenotypes^26^. Based on validation of mRNA expression data from the GSE7670 samples evaluated by GSEA, we found that PNO1 expression was positively correlated with EMT (Fig. 5c, d). We further evaluated the protein expression of each Notch intracellular domain (Notch1-4), Notch target genes (such as Hes5 and Hey1) and EMT related markers (E-cadherin, N-cadherin and vimentin). The results revealed that knocking down PNO1 expression suppressed the protein level of Notch2, Notch4 and Hey1 in both A549 and NCI-H1299 cell lines. PNO1 also induced changes in EMT-associated markers, including the upregulation of E-cadherin and downregulation of N-cadherin and vimentin (Fig. 5e). Nine markers in the Notch signaling pathway as Notch components were further validated by subsequent RT-PCR. The expression of Notch pathway ligands such as Notch2, Notch4, Delta-like 1 (DLL1) and Delta-like 4 (DLL4) was downregulated both in sh-PNO1 (A549 and NCI-H1299) groups (Fig. 5f, g). In addition, the epithelial-associated transcription factors expression (such as OVOL1 and OVOL2) was upregulated in sh-PNO1 (A549 and NCI-H1299) groups (Fig. 5h, i). The results of mesenchymal-associated transcription factors (such as N-cadherin, vimentin, ZEB1 and Twist) were reversed (Fig. 5h, i). We further explored the relationship between most of EMT markers mRNA expression and PNO1 expression (Supplementary Fig. 5A-D) in LUAD patients by cBioPortal (TCGA PanCancer Atlas). Some findings indicated that MK-0752 might be a pan-Notch inhibitor gamma secretase inhibitor (GSIs)^27^. To further confirmed the relationship between PNO1 and Notch signaling pathway, we detected the protein expression of each Notch intracellular domain (Notch1-4) or Notch target genes (Hes5 and Hey1) in MK-0752-treated PNO1 overexpression cells and control groups (Fig. 5j). Firstly, we detected the PNO1 expression in these three groups. The expression of PNO1 was increased in PNO1 and PNO1 + MK-0752 group. It demonstrated that MK-0752 could not influence the expression of PNO1. Furthermore, in Vector and PNO1 groups, PNO1 overexpression increased the protein level of Notch2, Notch4 and Hey1 in both A549 and NCI-H1299 cell lines. There was a potential difference of Hes5 expression in Vector and PNO1 groups of NCI-H1299 cell lines. When treated with MK-0752, the protein level of Notch2, Notch4 and Hey1 were obviously decreased. In these three groups, we further evaluated migration ability by wound-healing assay and proliferation potential by colony formation assay (Fig. 5k–n). Interestingly, MK-0752 treatment attenuated the process of migration (Fig. 5k, l) and growth (Fig. 5m, n) induced by PNO1 overexpression. In summary, we supposed that the inactivation of Notch signaling by a MK-0752 could reverse the EMT process for LUAD patients with PNO1 overexpression in the future.

### PNO1 promotes LUAD tumorigenesis in vivo

**Fig. 6:**
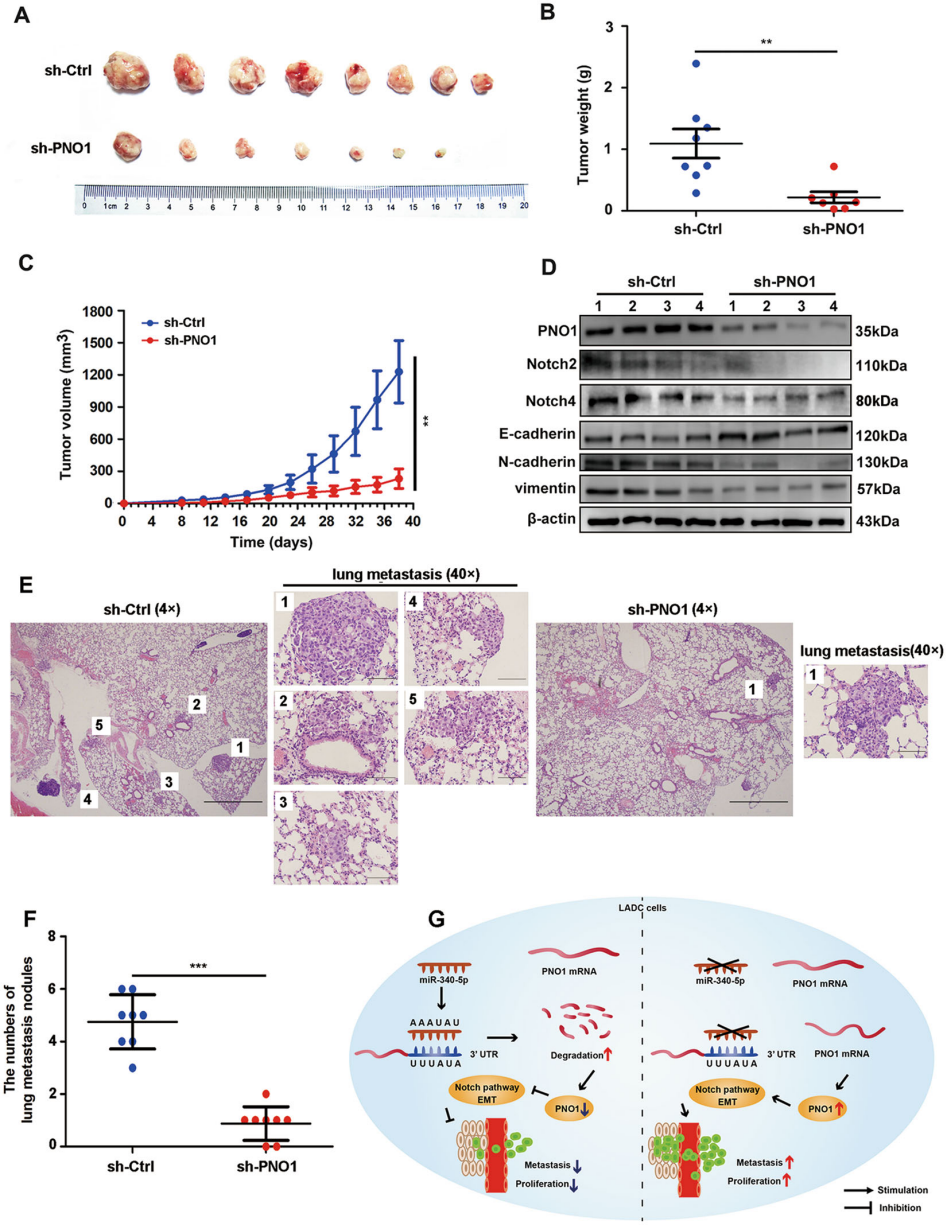
PNO1 promotes LUAD tumorigenesis in vivo. **a** Images of tumors from nude mice in the sh-Ctrl and sh-PNO1 groups. **b**, **c** Tumor weights and volumes in the two groups. *P < 0.05, **P < 0.01, ***P < 0.001. **d** The protein level of PNO1, EMT markers and Notch intracellular domains in established xenograft model assessed by Western Blotting. **e** H&E staining about lung metastasis of tail vein injected mice, 4× and 40×. **f** The numbers of lung metastasis nodules in sh-Ctrl and sh-PNO1 group. **g** A proposed mechanistic scheme for PNO1 regulated by miR-340-5p induced lung adenocarcinoma proliferation and metastasis through Notch pathway.

As PNO1 promotes the proliferation and metastasis of LUAD cells in vitro, we assumed that knocking down PNO1 expression might also suppress LUAD cell tumorigenesis in vivo. NCI-H1299 cells with stably downregulated PNO1 expression (sh-PNO1) or expression of a control vector (sh-Ctrl) were subcutaneously implanted into nude mice (5 × 10^6^/mice). We measured tumor volume every other day beginning at the second week after the first injection. In these two groups, all the mice were sacrificed at the end of the sixth week, and the primary tumors are shown in Fig. 6a. Tumor weights were significantly lower in the sh-PNO1 group than in the sh-Ctrl group (Fig. 6b). Tumor sizes were also much smaller in the sh-PNO1 group (Fig. 6c). We examined the expression of PNO1, EMT markers and Notch intracellular domains in our established xenograft model. According to the results of Fig. 5, we considered that downregulation of PNO1 inhibited the expression of Notch2 and Notch4. Thus, we further detected the expression of Notch2 and Notch4 in sh-Ctrl and sh-PNO1 group. The results were consistent with that in cell lines. Furthermore, we assessed the E-cadherin, N-cadherin, and vimentin expression in the established xenograft model. We found that PNO1 promoted epithelial mesenchymal transition of LUAD in vivo (Fig. 6d). We also established lung metastasis mouse models by tail vein injection. After six weeks, the mice were sacrificed and the lung were harvested. We found that PNO1 downregulation A549 cells formed fewer lung metastatic nodules by HE staining (Fig. 6e). The averages number of metastatic nodules per lung was 4.75 in mice injected with control cells but only 0.88 in mice injected with shPNO1 cells (Fig. 6f).

## Discussion

Several studies have revealed that ribosome biogenesis serves an essential role in cancer progression^28,29^. Ribosome biogenesis is a highly multiplexed process that is mainly regulated by approximately two hundred ribosome assembly factors^30^. By Northern blotting and RNA profiling, Peng^20^ confirmed that PNO1 played a critical role in the process of ribosome biogenesis in human CRC cells. Specifically, downregulation of PNO1 expression decreased the amounts of 18S rRNA, 40S subunits, 60S subunits and the 80S ribosome. These changes led to a significant inhibition of global protein synthesis. Wu^31^ revealed that PNO1 participated in promoting proliferation and migration of UBC cells. In addition, PNO1 KD attenuated the tumorigenesis potential of UBC in mouse. Pan^32^ offered the first evidence for a role of PNO1 as an HCC oncogene. Thus, further research may focus on the potential of PNO1 as a prognostic biomarker and the function of PNO1 as an oncogene in tumor progression.

NSCLC accounts for the majority of lung cancer cases, while LUAD accounts for approximately half of the NSCLC cases^33^. According to the heterogeneity of LUAD, it is still a substantial challenge to develop effective individualized treatments^34^. Thus, there is an urgent demand to explore novel and effective biomarkers for predicting the prognosis of LUAD. In our study, we found that PNO1 expression was upregulated in LUAD tissue samples and predicted poor survival in LUAD patients by analysis of TCGA and GEO datasets, RT-PCR and Western Blotting. Surprisingly, among NSCLC, LUAD and SCLC patients, only LUAD patients in the TCGA database showed a detectable correlation between PNO1 expression and prognosis. Furthermore, we confirmed that PNO1 served as an oncogene in most tumors (including LUAD and CRC tumors) by utilizing the pancancer view of UALCAN. These series of results demonstrated the probability that PNO1 can be used as a specific biomarker and target in LUAD.

Although both LUAD and SCLC are histologically classified as NSCLC, they differ greatly in molecular make-up, response to systemic treatment strategies and survival prognosis^35,36^. To verify the results for the TCGA dataset, we detected PNO1 expression in 120 LUAD and 107 SCLC patient samples using immunohistochemistry. Similarly, increased expression of PNO1 was an independent risk factor for LUAD but not for SCLC. Interestingly, similar to the adenoma-carcinoma evolution of CRC, the development of LUAD follows a linear multistep process^37^. In other words, the biological function of LUAD may be more similar to that of CRC than that of SCLC. Peng^20^ reported that PNO1 was highly expressed in CRC and associated with a poor prognosis. In conclusion, PNO1 serves as a specific biomarker in LUAD patients. More importantly, this is the first evidence of the significance of PNO1 in LUAD. We also found that PNO1 mRNA expression was positively correlated with lymph node metastasis (Supplementary Fig. 1D). But in our IHC data, we did not analyze the consistent correlation. The difference in the ratio of LN (+) patients might be the cause of the lack of correlation by IHC detection. And the IHC data mainly indicated the protein expression of PNO1 instead of mRNA expression (GEO database). But the overall trend (lymph-node metastasis and advanced TNM stage) of the two results was consistent. Furthermore, we also demonstrated the functional role of PNO1 in breast cancer. PNO1 may be used as a promising biomarker in breast cancer (unpublished data). Thus, we hypothesize that PNO1 may act as a potential prognostic marker in adenocarcinoma-like malignancies according to the findings for LUAD, breast cancer and CRC.

Our research demonstrated that downregulation of PNO1 expression suppressed LUAD cell migration and invasion. In addition, we confirmed that PNO1 promoted LUAD cell proliferation and inhibited cell apoptosis. Notably, these phenomena were observed in a xenograft animal model and lung metastasis assay. Thus, we initially ascertained the oncogenic functions of PNO1 in LUAD cells in vivo and in vitro. Based on the series of experiments, we hypothesized that PNO1 may serve as a potential target in LUAD patients.

miRNAs are short (~22 nucleotides), endogenous, noncoding RNA molecules that regulate gene expression^38^. In animals and plants, miRNAs play critical regulatory roles by targeting mRNAs for translational suppression or degradation^39^. miRNAs can serve as tumor suppressor genes and exert important inhibitory functions in carcinogenesis^40,41^. In our study, we confirmed the direct interaction between PNO1 and miR-340-5p. In addition, in contrast with the expression of PNO1, miR-340-5p expression was downregulated in LUAD tissue samples. Previous studies have demonstrated that miR-340-5p plays essential roles in tumor repression. miR-340-5p can suppress aggressiveness by targeting Bcl-w and Sox2 in glioblastoma multiforme (GBM)^25^. However, the functions of miR-340-5p in LUAD have not been clearly reported. Our research showed that miR-340-5p suppressed cell proliferation and metastasis in LUAD cells. More importantly, by rescue experiments, we verified that the functional roles of PNO1 could be regulated by miR-340-5p expression levels in LUAD. Taken together, our findings revealed that targeted regulation of PNO1 by miR-340-5p was a definite upstream molecular mechanism in LUAD. In the future, miR-340-5p may serve as an inhibitor to treat LUAD patients with PNO1 overexpression.

Several studies have confirmed that the Notch signaling pathway regulates cell differentiation, proliferation, apoptosis and metastasis in most of cancers such as lung cancer, CRC and breast cancer^26,27,42^. By GSEA datasets, Western Blotting and RT-PCR, the Notch components (such as Notch2, Notch4, and Hey1) expression was downregulated in sh-PNO1 groups. Several studies reported the important role of Nocth2, Nocth4 and Hey1 in lung cancer progress^43–46^. In addition, recent researches indicate that Notch signaling plays an essential role in lung cancer initiation and cross-talks with some transcriptional factors to trigger EMT, promoting the progression of NSCLC^26^. Through a series of experiments, we confirmed the hypothesis by Notch components and EMT markers expression in different PNO1 expression groups. Thus, inactivation of Notch signaling by a γ-secretase inhibitor (such as MK-0752, RO4929097)^47^ could reverse the EMT process for LUAD patients with PNO1 overexpression in the future. There were still several limitations in this part of the research. First, further molecular biological experiments were needed to explore the underlying mechanism (such as interaction) of the Notch and EMT signaling pathway activation induced by the upregulation of PNO1. Second, previous studies on PNO1 about LUAD progression especially the correlation with Notch pathway were very limited, which also restricted our exploration of the molecular mechanism to a certain extent. Thus, our findings might firstly provide a basis for the exploration of the downstream mechanism of PNO1 expression upregulation induced LUAD progression in further studies.

In summary, we demonstrate that high expression of PNO1 is related to the poor prognosis of LUAD by using the TCGA and GEO databases and IHC staining. PNO1 may serve as a specific prognostic biomarker in LUAD patients. This study is the first demonstration of the prognostic specificity of PNO1 in LUAD patients. Moreover, PNO1 promotes the proliferation and metastasis of LUAD cells in vivo and in vitro. Furthermore, we report that the functional role of PNO1 may be regulated by miR-340-5p expression levels in LUAD. The results of rescue experiments confirm that PNO1 was a direct functional target of miR-340-5p. Based on GSEA analysis, Western Blotting and RT-PCR, we confirm that PNO1 expression was related with Notch pathway and EMT (Fig. 6g). Our results highlight the potential value of miR-340-5p, Notch inhibitors and PNO1 as promising targets for the treatment of LUAD in the future.

## Materials and methods

### Patients and tissue specimens

Human LUAD, SCLC and adjacent nonmalignant tissue samples were obtained from the Tianjin Medical University Cancer Institute and Hospital (Tianjin, China) between January 2007 and December 2010. None of the patients had undergone adjuvant therapy such as radiotherapy or chemotherapy before surgical resection. All tissue samples were verified to be LUAD, SCLC or adjacent normal tissue by two independent pathologists, and the LUAD samples were staged based on the 8th edition of the American Joint Committee on Cancer (AJCC) Cancer Staging Manual. The study was consistent with the ethical guidelines of the Helsinki Declaration and approved by the Ethics Committee.

### Cell culture

A549 and NCI-H1299 cells were both purchased from the ATCC cell bank. Based on culture requirements (37 °C, 5% CO_2_), the cell lines were cultured in complete medium RPMI 1640 (Corning, USA) supplemented with 10% fetal bovine serum (FBS; PAN-Seratech) and 1% penicillin-streptomycin solution (PS; HyClone).

### RNA extraction, cDNA synthesis, and quantitative real-time PCR

Total RNA was isolated from adherent cells using Trizol reagent (Ambion, USA). Based on a quantitative real-time PCR (RT-PCR) kit (Takara, Japan), cDNA was synthesized by reverse transcription of the isolated RNA. The amplification reaction was conducted by using predesigned primers according to the manufacturer's instructions (Takara, Japan). For microRNA (miRNA) quantification, a miScript PCR system (Qiagen) was used, and reverse-transcription RT-PCR was carried out according to the manufacturer’s instructions. U6 small nuclear RNA (RNU6B; Qiagen) and β-actin served as endogenous controls. The primer sequences are shown in Supplementary Table 3.

### Western blotting and antibodies

The following antibodies were used: anti-PNO1 (1:1,000), anti-N-cadherin (1:500) from Santa Cruz Biotechnology, anti-E-cadherin (1:500) from BD Biosciences, anti-vimentin (1:4000) from Epitomics, anti-Nocth3 (1:1000) from Abcam (Cambridge, UK), anti-Nocth1 (1:1000), anti-Nocth2 (1:1000), anti-Nocth4 (1:1000), anti-β-actin (1:1,000) from Cell Signaling Technology (Beverly, Massachusetts) and Hes5 (1:800), Hey1 (1:500) from Bioss. After surgery, freshly isolated LUAD samples and adjacent nonmalignant tissue samples were frozen in liquid nitrogen, and the tissue proteins were extracted with a tissue protein extractor (Servicebio). On the other hand, cells were washed in cold PBS (pH 6.8) three times and lysed on ice for 30 min in an SDS lysis buffer supplemented with 1 mM NaF, 1 mM Na_3_VO_4_ and a 1× protease/phosphatase inhibitor cocktail (Roche, Switzerland). Protein denaturation was carried out at 95 °C for 10 min, followed by centrifugation at 12,000 rpm and 4 °C for approximately 10 min. Equal amounts of protein (30–60 μg) were loaded onto a gel and separated by SDS-PAGE. Then, the protein was transferred to a PVDF membrane (Immobilon-P; Millipore, Billerica, MA, USA), which was blocked with 5% skim milk or 3% bovine serum albumin (BSA). The membranes were blotted with primary antibodies overnight at 4 °C. A secondary antibody (1:4000) was incubated for 1 h at room temperature. The original data of all Western blots were shown in Supplementary Fig. 6.

### Statistical analyses

The clinical data were evaluated using SPSS 25.0 for Windows (SPSS Inc., Chicago, IL). The univariate Kaplan-Meier method and multivariate Cox method were used to analyze the independent risk factors and survival curves of LUAD, SCLC and NSCLC patients. In addition, based on survival information from the TCGA database, the cutoff value for PNO1 was set by the quartile method for further survival analysis. Spearman correlation analysis was used to analyze the relationships between the staining score of PNO1 and clinicopathological factors. The relationship between PNO1 and miR-340-5p was also evaluated by Spearman correlation analysis. An unpaired t-test or one-way ANOVA was used to analyze the expression of PNO1 or miR-340-5p between the tumor and paratumor tissues of LUAD patients.

The details about other assays were described in the Supplementary Materials and Methods.

## Supplementary information

Supplementary information

Supplementary Figure1

Supplementary Figure2

Supplementary Figure3

Supplementary Figure4

Supplementary Figure5

Supplementary Figure6

Supplementary Table 1

Supplementary Table 2

Supplementary Table 3
