# Supplementary material for: PNO1, which is negatively regulated by miR-340-5p, promotes lung adenocarcinoma progression through Notch signaling pathway
Source: Oncogenesis. 2020 Jun 1;9(5):58. doi: 10.1038/s41389-020-0241-0 (PMC7264314; doi:10.1038/s41389-020-0241-0)
Supplement: Supplementary file 1 — Supplementary information [file 41389_2020_241_MOESM1_ESM.docx]

**Supplementary information**

**Supplementary Materials and Methods**

**Cell transfection**

To obtain lentiviral particles, packaging plasmids (VSVG and ΔR) and expression plasmids (sh-Ctrl/Vector and sh-PNO1/PNO1) were transfected into HEK293T cells using Lipofectamine 2000 (Invitrogen). The HEK293T cells were used to produce the lentiviruses. A549 and NCI-H1299 cells were prepared for transfection. Polybrene (Solarbio) was used as the transfection reagent. A stably transfected cell line was obtained under puromycin (Gibco) selection.

To regulate miR-340-5p expression, cells were transient transfected by miRNA plasmid (RiboBio). In rescue experiments, cells were stably transfected with LV-hsa-miR-340-5p-inhibitor (45700-1), LV-hsa-miR-340-5p (45706-1) or a corresponding control lentivirus (GeneChem, Shanghai). The volume of lentivirus used for transfection was determined according to the multiplicity of infection (MOI), number of cells and titer of the lentivirus. HiTransG P (GeneChem, Shanghai) transfection reagent was used to improve the efficiency of transfection according to the manufacturer's recommendations. Stably transfected cell lines were obtained under neomycin (G418, Solarbio) selection. All transfections were conducted for 24-48 h.

**Immunohistochemistry staining**

IHC staining was used to examine the expression level of PNO1 in paraffin-embedded samples of LUAD and SCLC tissues according to previously described methods. An anti-PNO1 antibody was purchased from LSBio (USA, catalog no. LS-C179090, 1:200). The IHC score was used to evaluate the correlation between PNO1 expression and overall survival (OS) in LUAD and SCLC patients. Among the LUAD patients, the IHC score was also used in correlation analyses for different clinicopathological factors. The PNO1 staining intensity was scored across a range of four grades (0, no immune response; 1, weak immune response; 2, moderate immune response; and 3, strong immune response). The percentage of cells positive for PNO1 expression was assessed across a range from 0 to 3 (0, no positive cells; 1, < 30% positive cells; 2, 30-60% positive cells; and 3, 60%-100% positive cells). The IHC staining intensity score and percentage score were combined to obtain the final IHC score.

**Cell proliferation (MTT assay and CCK-8 assay)**

MTT assay: Initially, LUAD (A549 and NCI-H1299) cells were seeded in 96-well plates at a density of 2 × 10^3^ cells with 100 μL of complete medium RPMI 1640 per well. Three parallel wells were set up for each group. After one day, 20 μL of 3-(4,5-dimethylthiazol-2-yl)-2,5-diphenyl-2H-tetrazolium bromide (MTT; 5 mg/mL in PBS, Sigma, St. Louis, USA) were added to each well and incubated at 37 °C in 5% CO_2_ for 4 h, followed by medium removal and the addition of 100 μL of dimethyl sulfoxide (DMSO; Sigma). The optical density (OD) at a wavelength of 490 nm was measured using a microplate autoreader (Bio-Rad). Cell viability was examined at 24, 48, 72, 96 and 120 h.

Cell Counting Kit-8 (CCK-8) assay: For the CCK-8 assay, LUAD cells were plated in a 96-well plate at a density of 2 × 10^3^ cells per well. Six parallel wells were set up for each group. Immediately, 10 μL of CCK-8 (Dojindo Laboratories) reagent were added to each well and incubated with the cells for 4 h at 37 °C in 5% CO_2_. The OD value at a wavelength at 450 nm was measured by an enzyme-labeling instrument. Cell viability was examined at 0, 24, 48 and 72 h.

**Colony formation assay**

For cell survival analysis, sh-PNO1- and sh-Ctrl-transfected LUAD cells were plated in 6-well plates (1× 10^3^ cells/well) and incubated at 37 °C in 5% CO_2_ for 2 weeks. When colonies were visible, the cells were stained with crystal violet. The colonies were counted, and images were obtained by using a digital camera.

**Apoptosis analysis**

Cells were washed in ice-cold PBS followed by 200 μL of 1 × binding buffer and then stained for 15 minutes in the dark with Annexin V-APC at room temperature (eBioscience, USA). According to the number of cells, 400-800 μL of 1 × binding buffer was added, and then the cells were analyzed. The percentage of apoptotic cells was analyzed by the Guava easyCyte HT (Millipore).

**Wound healing assay**

Cells were seeded in 6-well plates at a density of 2× 10^6^ cells per well. The next day, a uniform wound was generated along the cells in the petri dishes by using a 2.5-μL pipette tip. Then, the cells were washed in PBS three times, and 2% FBS was added to the RPMI 1640 medium. The distance of the wound was recorded and evaluated by subtracting the distance between the edges measured at the appropriate time (3, 6, 9, 12, or 24 h) from the average distance measured at six random positions at 0 h. Images were captured at 0 and 24 h with a microscope at 10 × magnification. Data are shown as the mean ± SD.

**Migration assay**

For a migration assay, RPMI 1640 medium supplemented with 20% FBS was loaded in the lower chamber of a Transwell system, and (1-2) ×10^5^ cells in 2% FBS RPMI 1640 medium were plated on the 8-μm polyvinyl pyrrolidone-free polycarbonate filter membrane (Corning, Falcon^®^ Cell Culture Inserts). After incubating for 16-18 h at 37 °C in 5% CO_2_, the migrated cells on the bottom were fixed with 4% paraformaldehyde (PFA) for 20 minutes and stained. Then, the cells were counted by light microscopy.

**Invasion assay**

For an invasion assay, Matrigel-coated Transwell chambers were incubated in 24-well plates for more than 1 h at 37 °C. RPMI 1640 medium supplemented with 20% FBS was loaded in the lower chamber, and (1-2) ×10^5^ cells in 2% FBS RPMI 1640 medium were added to the upper chamber. After 16-18 h of incubation at 37°C in 5% CO_2_, the invaded cells on the bottom were fixed with 4% PFA for 20 minutes, stained, and counted by light microscopy.

**Luciferase reporter** **assays**

According to a dual-luciferase reporter assay system protocol, luciferase reporter assays were performed. The PNO1 3' untranslated region (UTR) and mutant UTRs were cloned into pmiR-RB-Report™ h-PNO1-WT and pmiR-RB-Report™ h-PNO1-MUT (RiboBio). HEK293T cells were incubated at 37 °C in 5% CO_2_. The HEK293T cells were cotransfected with the pmiR-PNO1 wild-type or mutant plasmid and miR-340-5p mimics or negative controls (NC) in a 96-well plate. Lipo6000TM (Beyotime Biotechnology) transfection reagent was used. Luciferase activity was measured at 24 h after transfection using the Dual-Glo® Luciferase Assay System (Promega) according to the manufacturer's instructions.

**miRNA Sensor Luciferase reporter assays**

The A549 cells were cotransfected with the pmiRS-RB-has-miR-340-5p Sensor and micrONTM hsa-miR-340-5p mimic (micrONTMmiRNA mimic Negative Control) or micrOFFTM hsa-miR-340-5p inhibitor (micrOFFTMmiRNA inhibitor Negative Control) in a 96-well plate. Lipofectamine TM 3000 (Invitrogen) transfection reagent was used. Luciferase activity was measured at 48 h after transfection using the Dual-Glo® Luciferase Assay System (Promega) according to the manufacturer's instructions.

**TCGA and GEO datasets**

Raw data from the TCGA (National Cancer Institute) and GEO (NCBI) databases related to LUAD, SCLC and NSCLC were downloaded from the official websites. Then, the data were normalized using R Studio. We analyzed the expression of PNO1 and miR-340-5p in matched tumor tissue and adjacent nonmalignant tissue samples from the TCGA mRNA and miRNA database. Further survival analysis relating the expression level of PNO1 with the prognosis of LUAD, SCLC and NSCLC patients was performed. The GEO database (GSE40791, GSE7670, GSE33532, GSE101929, GSE32863, GSE10072, GSE21933, GSE68571 and GSE27486) was also used to verify the expression levels of PNO1 and miR-340-5p in LUAD tissue samples.

**Gene Set Enrichment Analysis**

Gene set enrichment analysis (GSEA) was used to explore whether the PNO1 mRNA level was related to biological features of LUAD, including tumor metastasis, tumor proliferation and patient survival status, on the basis of the GSE40791 and GSE7670 dataset for LUAD by GSEA 4.0.0 (The Broad Institute of MIT and Harvard).

**miRNA-mRNA interactions**

TargetScan (http://www.targetscan.org/vert_72/) and miRDB (<http://www.mirdb.org/>) were used to predict the potential upstream miRNAs that regulated PNO1.

**Chemicals**

Gamma secretase inhibitor MK-0752 was purchased from Selleck and dissolved in DMSO to make a 10 mM stock solution. The effective concentration of MK-0752 was 50 μM for *in vitro* cell culture.

***In vivo* experiments**

Animal experiments were approved by the Ethics Committee of the Tianjin Medical University Cancer Institute and Hospital. Five-week-old nude mice (SPF Biotechnology Co., Ltd, Beijing, China) were purchased for xenograft animal experiments (n = 8 per group). sh-Ctrl/NCI-H1299 and sh-PNO1/NCI-H1299 cells were prepared, and 5 × 10^6^ cells in 100 μL of PBS were injected subcutaneously. Tumor volume was monitored every two days using a vernier caliper. To generate lung metastasis models, the sh-Ctrl/A549 and sh-PNO1/A549 cells were prepared (8 mice/group, 2 × 10^6^/mL; 100μL per mice) were injected into the tail vein. After six weeks, the mice were sacrificed and the lung were harvested. The harvested lung tissues were formalin-fixed and paraffin-embedded. The tissues were subjected to H&E staining. A method of randomization was performed to determine how animals were assigned to experimental groups and processed, and the investigator was blinded to the group assignment during the assays. All animal protocols were approved by the ethics committee of the Tianjin Medical University Cancer Institute and Hospital and the disposal methods followed the animal ethics standards.

**Supplementary figure legends**

**Supplementary Figure. 1.**

(A). TCGA and GEO (GSE40791, GSE7670, GSE33532, GSE101929, GSE32863, GSE10072 and GSE21933) exploration of the expression of PNO1 in LUAD tissue samples. (B, C). TCGA and GSE40791 exploration of the expression of PNO1 in different TNM stage patients. (D). GSE68571 exploration of the expression of PNO1 in LUAD patients with different LN metastasis status. (E-G). Kaplan-Meier survival curve analysis of TCGA and GSE68571 data to validate the correlation between the expression of PNO1 and LUAD patient OS or DFS. (H, I). GSEA validating the correlation between PNO1 expression and survival in LUAD patients. (J). GSEA exploring the correlation between PNO1 expression and chemotherapy resistance. (K). GSEA of the relationship between the expression of PNO1 and tumor proliferation. (L, M). GSEA of the relationship between the expression of PNO1 and tumor metastasis. ^ns^*P* > 0.05, **P* < 0.05, ***P* < 0.01, ****P* < 0.001.

**Supplementary Figure. 2**

(A). TCGA exploration of the expression of PNO1 in SCLC and adjacent nonmalignant tissue samples. (B, C). Kaplan-Meier survival curve analysis of TCGA data to validate the correlation between the expression of PNO1 and SCLC patient OS or DFS. (D). TCGA exploration of the expression of PNO1 in NSCLC and adjacent nonmalignant tissue samples. (E, F). Kaplan-Meier survival curve analysis of TCGA data to validate the correlation between the expression of PNO1 and NSCLC patient OS or DFS. (G). Pancancer view in UALCAN showing the expression of PNO1 in most cancers. (H). The survival curve about PNO1 expression and LN (+) patients. **P* < 0.05, ***P* < 0.01, ****P* < 0.001.

**Supplementary Figure. 3**

(A, B). The construction of PNO1-control (Vector) and PNO1-overexpression (PNO1) A549 or NCI-H1299 LUAD cell lines. (C, D). The chemotaxis potential of the A549 and NCI-H1299 Vector and PNO1 groups (scale bar, 1.0 mm). (E, F). Wound-healing assay comparing the migration distance between the PNO1 and Vector groups of A549 or NCI-H1299 cells. (G, H). CCK-8 assay showing the proliferative abilities in the Vector and PNO1 groups of A549 or NCI-H299 cells. (I). The representative graphs of Annexin V/PI assay in sh-Ctrl and sh-PNO1 group. (J). The analysis of early and late apoptosis in sh-Ctrl and sh-PNO1 group. **P* < 0.05, ***P* < 0.01, ****P* < 0.001.

**Supplementary Figure. 4**

(A). Pattern diagram for predicting potential miRNAs upstream of PNO1. (B, C). The potential miRNAs of intersection determined by TargetScan and miRDB. (D-F). TCGA exploration of the expression of miR-377-3p, miR-6504-5p and miR-3064-3p in LUAD and adjacent nonmalignant tissue samples. (G, H). Wound-healing assay showing the different migratory abilities of A549 and NCI-H1299 cells with different expression levels of miR-340-5p. (I, J). Colony formation assay showing the different proliferative potentials of A549 and NCI-H1299 cells with different expression levels of miR-340-5p. (K, L). The expression of miR-340-5p and PNO1 in Control miR+Vector, miR-340-5p+Vector and miR-340-5p+PNO1 groups of A549 cell line. (M, N) Different proliferative potentials and migratory abilities associated with different expression levels of miR-340-5p and PNO1 in A549 cell line. ^ns^*P* > 0.05, **P* < 0.05, ***P* < 0.01, ****P* < 0.001.

**Supplementary Figure. 5**

(A-D). The correlation analysis of EMT markers and PNO1 expression in LUAD patients by cBioPortal (TCGA PanCancer Atlas: ‘mRNA expression, RSEM (Batch normalized from Illumina HiSeq_RNASeqV2’).

**Supplementary Figure. 6**

The original data of all Western blots.

**Supplementary table legends**

**Supplementary Table 1.**

Univariate and multivariate analysis of prognostic factors associated with OS in 120 LUAD patients

**Supplementary Table 2.**

Relationship between clinicopathological characteristics and PNO1 expression in 120 LUAD patients

**Supplementary Table 3.**

RT-PCR primers of mRNA and miRNA
