# Supplementary figures and images for: PNO1, which is negatively regulated by miR-340-5p, promotes lung adenocarcinoma progression through Notch signaling pathway
Source: Oncogenesis. 2020 Jun 1;9(5):58. doi: 10.1038/s41389-020-0241-0 (PMC7264314; doi:10.1038/s41389-020-0241-0)

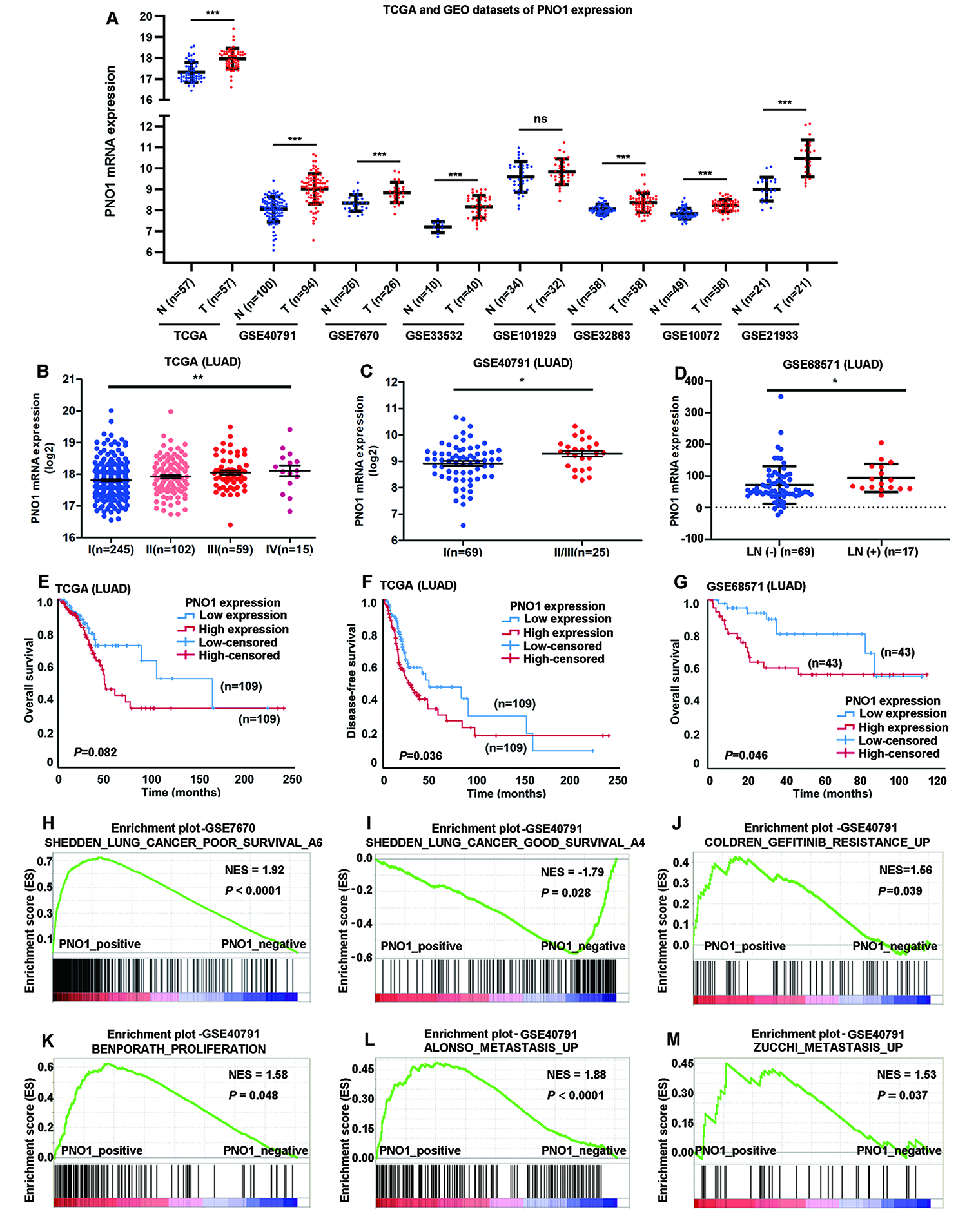

Supplement: Supplementary file 2 — Supplementary Figure1 [file 41389_2020_241_MOESM2_ESM.tif]

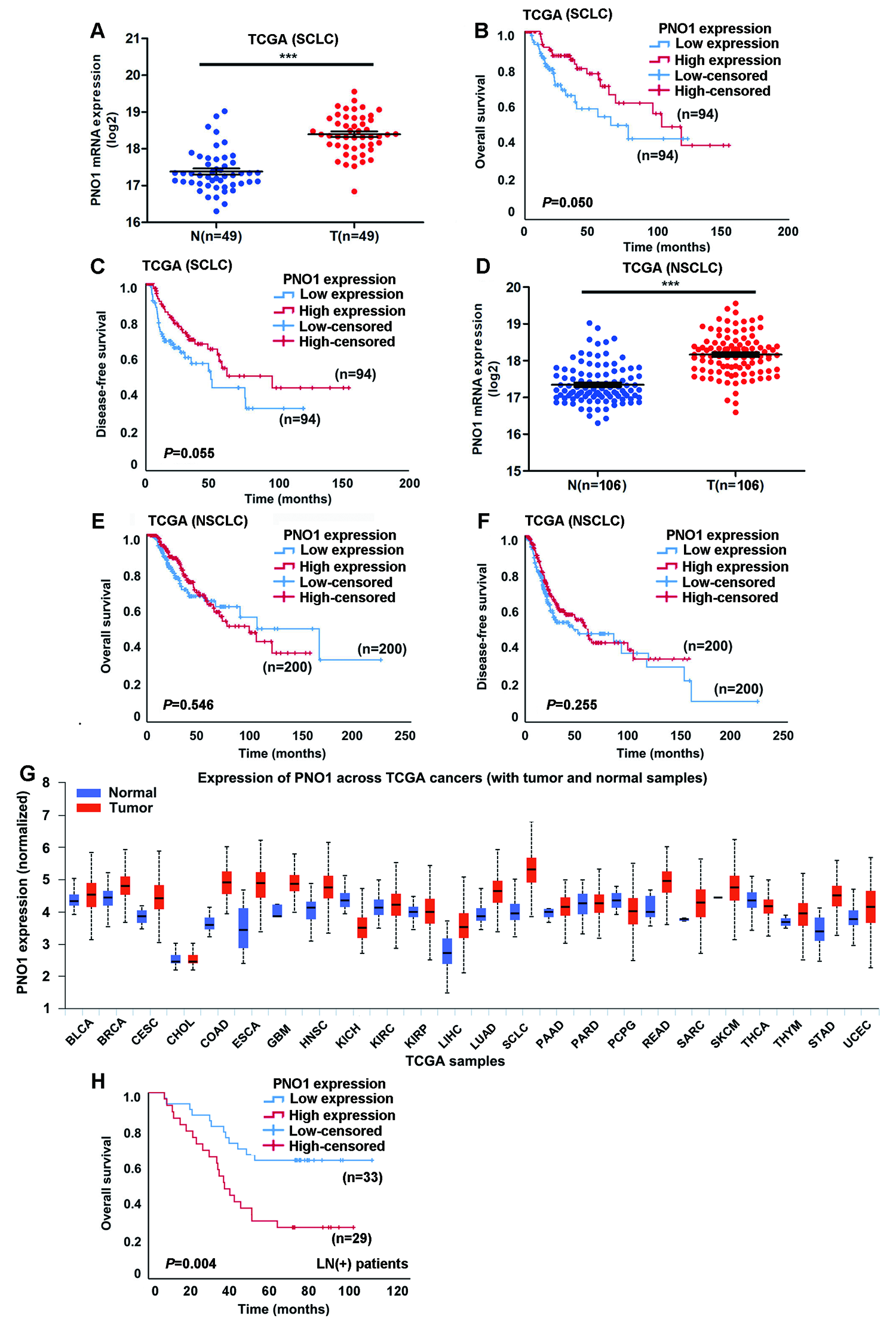

Supplement: Supplementary file 3 — Supplementary Figure2 [file 41389_2020_241_MOESM3_ESM.tif]

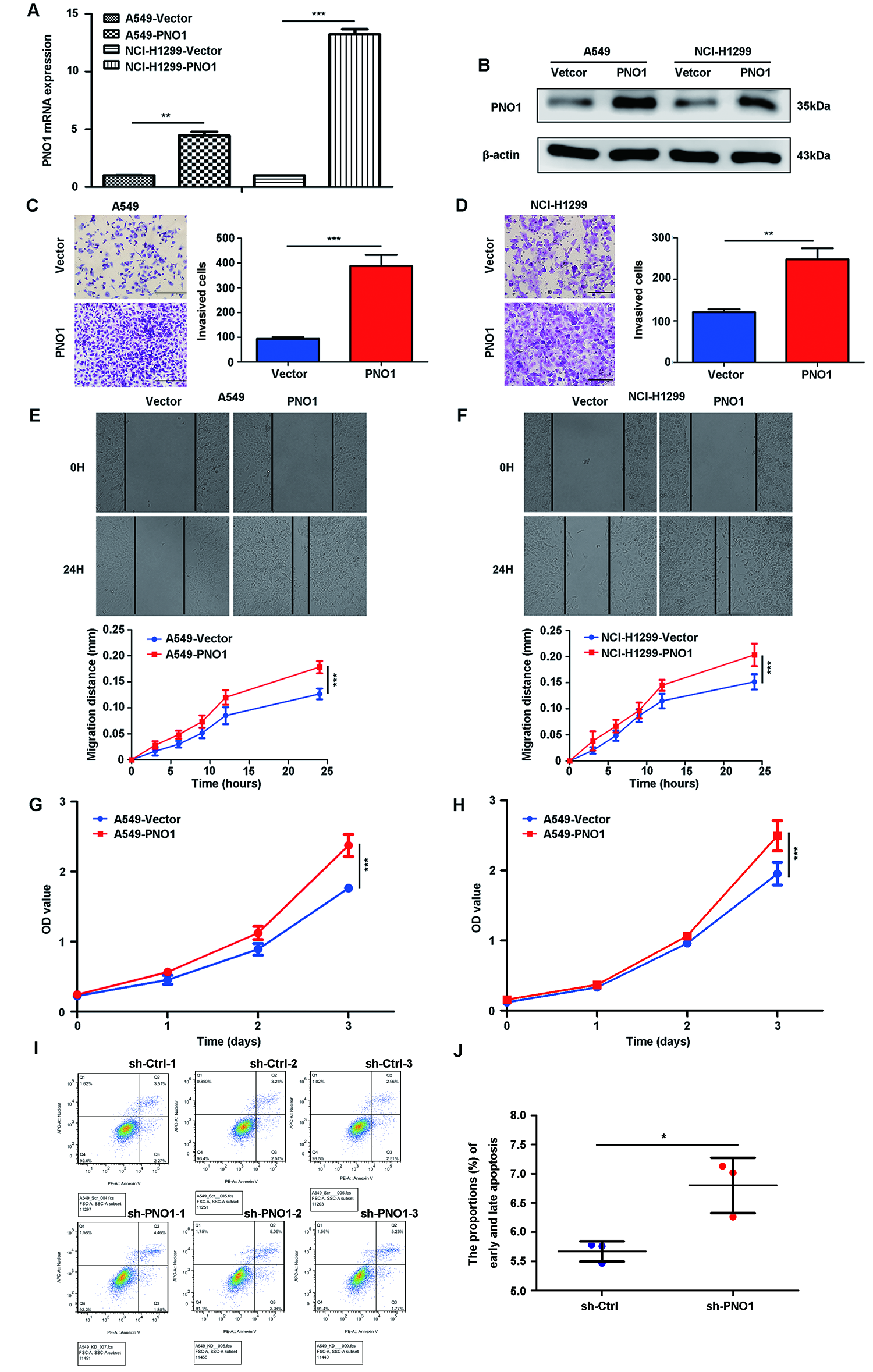

Supplement: Supplementary file 4 — Supplementary Figure3 [file 41389_2020_241_MOESM4_ESM.tif]

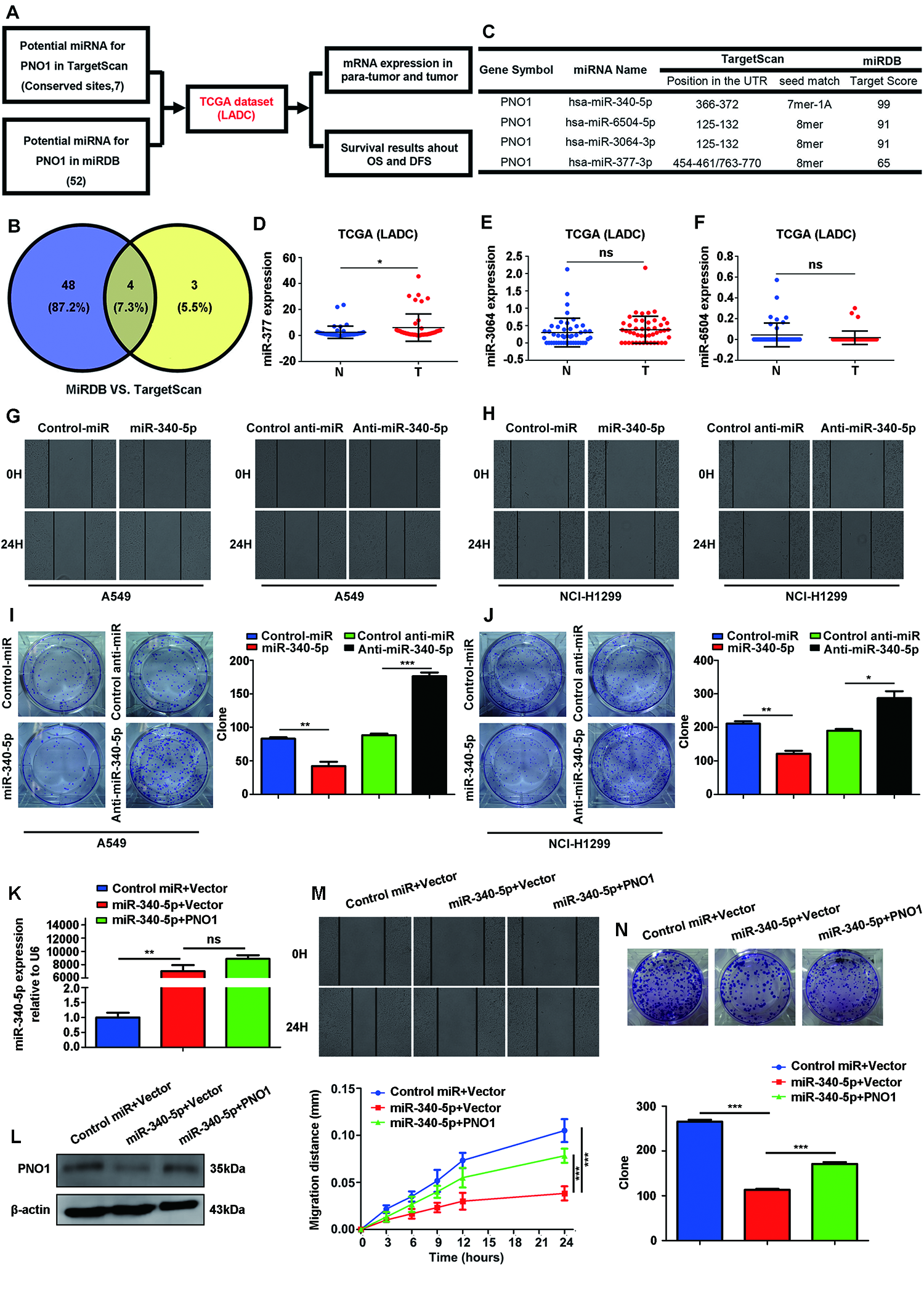

Supplement: Supplementary file 5 — Supplementary Figure4 [file 41389_2020_241_MOESM5_ESM.tif]

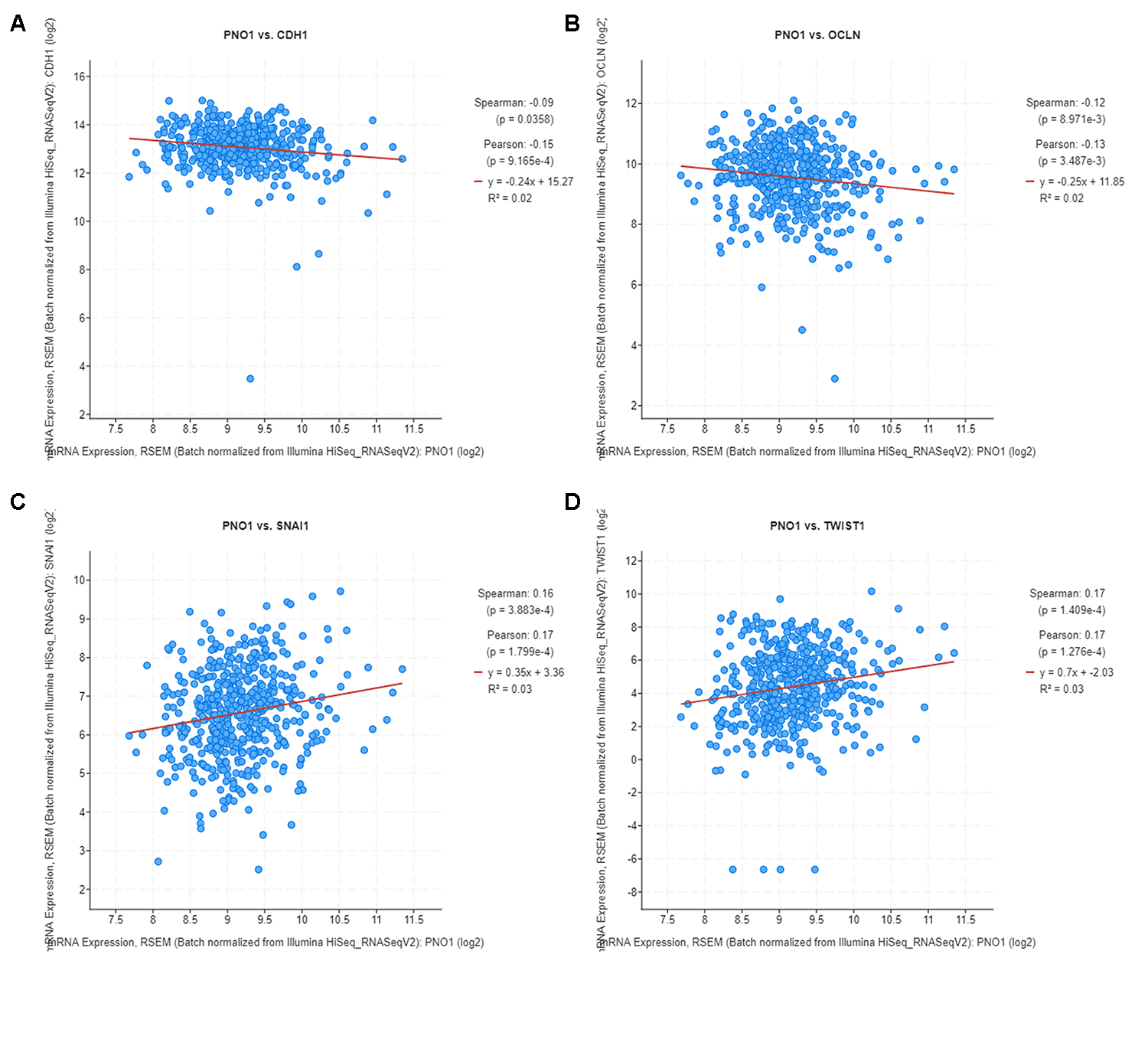

Supplement: Supplementary file 6 — Supplementary Figure5 [file 41389_2020_241_MOESM6_ESM.tif]

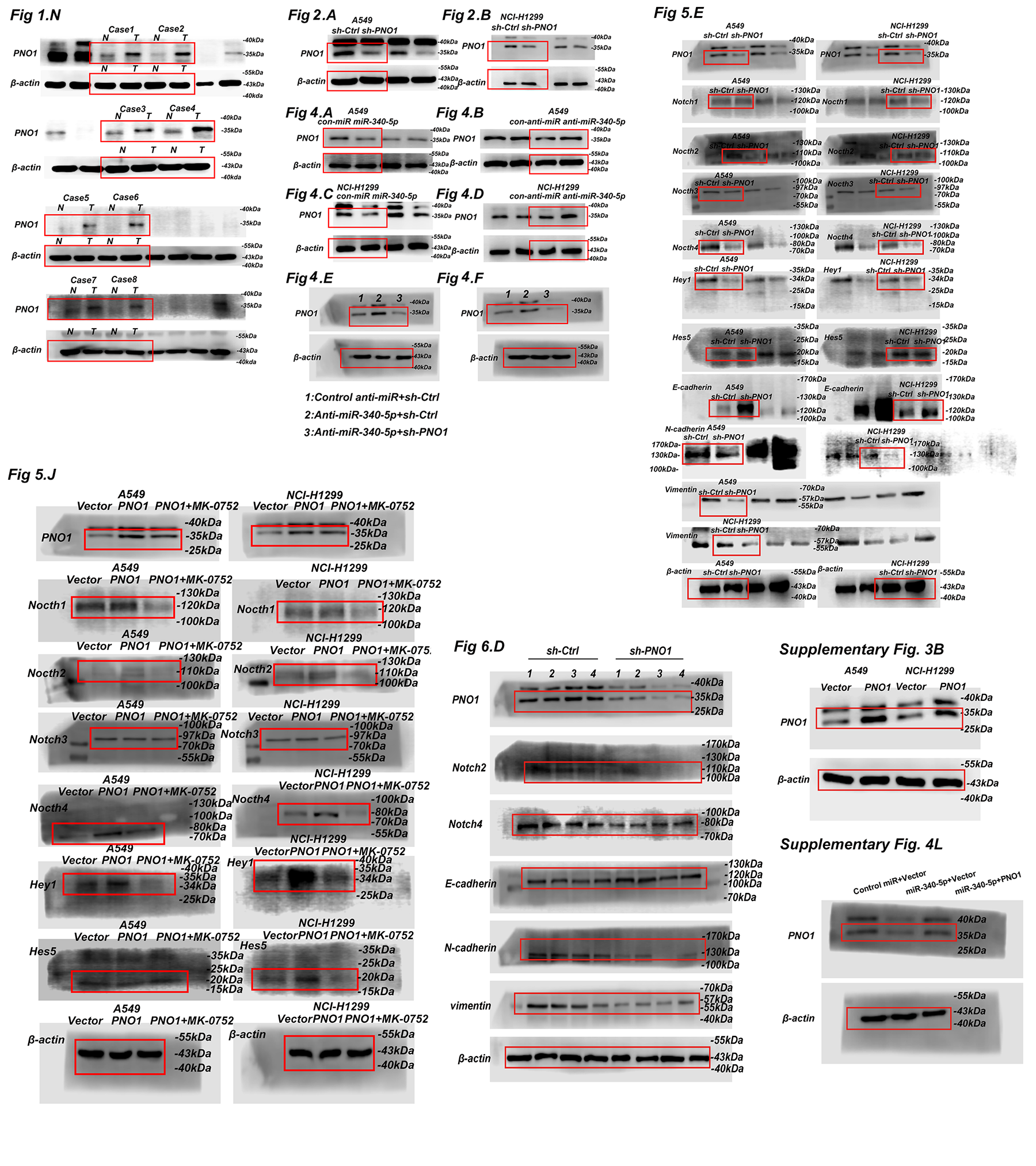

Supplement: Supplementary file 7 — Supplementary Figure6 [file 41389_2020_241_MOESM7_ESM.tif]
