# Supplementary material for: PNO1, which is negatively regulated by miR-340-5p, promotes lung adenocarcinoma progression through Notch signaling pathway
Source: Oncogenesis. 2020 Jun 1;9(5):58. doi: 10.1038/s41389-020-0241-0 (PMC7264314; doi:10.1038/s41389-020-0241-0)
Supplement: Supplementary file 8 — Supplementary Table 1 [file 41389_2020_241_MOESM8_ESM.docx]

| **Supplementary Table 1. Univariate and multivariate analysis of prognostic factors associated with OS in**  **120 LUAD patients** | | | | | |
| --- | --- | --- | --- | --- | --- |
|  |  |  |  |  |  |
|  |  |  |  |  |  |
| **LUAD patients (n=120)** | **Number** | **Univariate Analysis** | | **Multivariate Analysis** | |
|  |  | **5-year OS (%)** | ***P*-value** | **HR (95% CI)** | ***P*-value** |
| **Age(years)** ＞55/≤55 | 81/39 | 63.0/53.8 | 0.325 |  |  |
| **Sex** male/female | 56/64 | 62.5/57.8 | 0.505 |  |  |
| **Smoking history** Y/N | 60/60 | 63.3/56.7 | 0.395 |  |  |
| **Tumor size(cm)** ＞3/≤3 | 68/52 | 57.4/63.5 | 0.404 |  |  |
| **LN metastasis** Y/N | 62/58 | 46.8/74.1 | **0.002*** | 2.455(1.118,5.391) | **0.025*** |
| **TNM Stage** I/II/III | 37/36/47 | 75.7/58.3/48.9 | **0.042*** | 1.074(0.665,1.734) | 0.770 |
| **Staining score of PNO1** ≥1/＜1 | 53/67 | 49.1/68.7 | **0.026*** | 1.915(1.065,3.442) | **0.030*** |
