# Supplementary material for: PNO1, which is negatively regulated by miR-340-5p, promotes lung adenocarcinoma progression through Notch signaling pathway
Source: Oncogenesis. 2020 Jun 1;9(5):58. doi: 10.1038/s41389-020-0241-0 (PMC7264314; doi:10.1038/s41389-020-0241-0)
Supplement: Supplementary file 9 — Supplementary Table 2 [file 41389_2020_241_MOESM9_ESM.docx]

| **Supplementary Table 2. Relationship between clinicopathological characteristics and PNO1 expression in**  **120 LUAD patients** | | | | | | | | | | | |
| --- | --- | --- | --- | --- | --- | --- | --- | --- | --- | --- | --- |
|  |  |  |  |  |  |  |  |  |  |  |  |
| **Characteristics** |  | **Total** | **PNO1 expression** | | ***P*-value** | **Characteristics** | | **Total** | **PNO1 expression** | | ***P-*value** |
|  |  | **120** | **Low** | **High** |  |  |  | **120** | **Low** | **High** |  |
| **Age (years)** |  |  |  |  | 0.930 | **Size (cm)** |  |  |  |  | **0.009*** |
|  | ＞55 | 81 | 45 | 36 |  |  | ＞3 | 68 | 31 | 37 |  |
|  | ≤55 | 39 | 22 | 17 |  |  | ≤3 | 52 | 36 | 16 |  |
| **Sex** |  |  |  |  | 0.172 | **LN metastasis** |  |  |  |  | 0.556 |
|  | Male | 56 | 35 | 21 |  |  | Present | 62 | 33 | 29 |  |
|  | Female | 64 | 32 | 32 |  |  | Absent | 58 | 34 | 24 |  |
| **Smoking history** |  |  |  |  | 0.201 | **TNM stage** |  |  |  |  | **0.014*** |
|  | Present | 60 | 37 | 23 |  |  | I stage | 37 | 22 | 15 |  |
|  | Absent | 60 | 30 | 30 |  |  | II stage | 36 | 28 | 8 |  |
|  |  |  |  |  |  |  | III stage | 47 | 17 | 30 |  |
