# Supplementary material for: PNO1, which is negatively regulated by miR-340-5p, promotes lung adenocarcinoma progression through Notch signaling pathway
Source: Oncogenesis. 2020 Jun 1;9(5):58. doi: 10.1038/s41389-020-0241-0 (PMC7264314; doi:10.1038/s41389-020-0241-0)
Supplement: Supplementary file 10 — Supplementary Table 3 [file 41389_2020_241_MOESM10_ESM.docx]

| **Supplementary Table 3. RT-PCR primers of mRNA and miRNA** | |
| --- | --- |
| **mRNA** | **primer sequences** |
| Human PNO1 Forward Primer | 3’-TGTTAAACCCCTAAAGGGAGACC-5’ |
| Human PNO1 Reverse Primer | 5’-CCTTGTCCGTGTCACATTCTCT-3’ |
| Human β-actin Forward Primer | 3’-TCATCACCATTGGCAATGAG-5’ |
| Human β-actin Reverse Primer | 5’-CACTGTGTTGGCGTACAGGT-3’ |
| Human E-cadherin Forward Primer | 3’-GTCACTGACACCAACGATAATCCT-5’ |
| Human E-cadherin Reverse Primer | 5’-TTTCAGTGTGGTGATTACGACGTTA-3’ |
| Human occludin Forward Primer | 3’-CACACTTGCTTGGGACAGAGG-5’ |
| Human occludin Reverse Primer | 5’-TGAGCCGTACATAGATCCAGGAGC-3’ |
| Human OVOL1 Forward Primer | 3’-CACGTGCAAGAGGAACTGGA-5’ |
| Human OVOL1 Reverse Primer | 5’-GTTCGGCCACAGAGGGTT-3’ |
| Human OVOL2Forward Primer | 3’-TCACCTCAAGTGCCACAACCA-5’ |
| Human OVOL2 Reverse Primer | 5’-TGTAGCCGCAATCCTCGCA-3’ |
| Human N-cadherin Forward Primer | 3’-AGCCAACCTTAACTGAGGAGT-5’ |
| Human N-cadherin Reverse Primer | 5’-GGCAAGTTGATTGGAGGGATG-3’ |
| Human vimentin Forward Primer | 3’-GATGCCCTTAAAGGAACCAATGAG-5’ |
| Human vimentin Reverse Primer | 5’-GGCGGCCAATAGTGTCTTGGTAG-3’ |
| Human ZEB1 Forward Primer | 3’-TTCAAACCCATAGTGGTTGCT-5’ |
| Human ZEB1 Reverse Primer | 5’-TGGGAGACACCAAACCAACTG-3’ |
| Human Twist Forward Primer | 3’-GGAGTCCGCAGTCTTACGAG-5’ |
| Human Twist Reverse Primer | 5’-TCTGGAGGACCTGGTAGAGG-3’ |
| Human Slug Forward Primer | 3’-TACCGCTGCTCCATTCCACG-5’ |
| Human Slug Reverse Primer | 5’-CATGGGGGTCTGAAAGCTTGG-3’ |
| Human Snail Forward Primer | 3’-CTGGGTGCCCTCAAGATGCA-5’ |
| Human Snail Reverse Primer | 5’-CCGGACATGGCCTTGTAGCA-3’ |
| Human Notch1 Forward Primer | 3’-GCTGCCTCTTTGATGGCTTCGA-5’ |
| Human Notch1 Reverse Primer | 5’-CACATTCGGCACTGTTACAGCC-3’ |
| Human Notch2 Forward Primer | 3’-TGGGCTACACTGGGAAAAAC-5’ |
| Human Notch2 Reverse Primer | 5’-ACATAGGCACTGGGACTCTG-3’ |
| Human Notch3 Forward Primer | 3’-TCTTGCTGCTGGTCATTCTC-5’ |
| Human Notch3 Reverse Primer | 5’-TGCCTCATCCTCTTCAGTTG-3’ |
| Human Notch4 Forward Primer | 3’-AGTCCAGGCCTTGCCAGAACG-5’ |
| Human Notch4 Reverse Primer | 5’-GTAGAAGGCATTGGCCAGAGAG-3’ |
| Human Jag1 Forward Primer | 3’-AGAAGTCAGAGTTCAGAGGCGTCC-5’ |
| Human Jag1 Reverse Primer | 5’-AGTAGAAGGCTGTCACCAAGCAAC-3’ |
| Human Jag2 Forward Primer | 3’-AACGATACCACCCCGAATGAGG-5’ |
| Human Jag2 Reverse Primer | 5’-GCTGCCACAGTAGTTCAGGTCTTTG-3’ |
| Human DLL1 Forward Primer | 3’-ATCTGCCTGCCTGGATGTGATG-5’ |
| Human DLL1 Reverse Primer | 5’-AGACAGCCTGGATAGCGGATACAC-3’ |
| Human DLL3 Forward Primer | 3’-CAATGGAGGCAGCTGTAGTG-5’ |
| Human DLL3 Reverse Primer | 5’-TCAAAGGACCTGGGTGTCTC-3’ |
| Human DLL4 Forward Primer | 3’-TTGGATGAGCAAACCAGCACCC-5’ |
| Human DLL4 Reverse Primer | 5’-TGACAGCCCGAAAGACAGATAGG-3’ |
| **miRNA** |  |
| has-miR-340-5p | 5'-UUAUAAAGCAAUGAGACUGAUU-3' |
